# Nitrite Reduction at Low Overpotentials on N‑Doped Carbon: When Metal Single Atoms Become Poisons

**DOI:** 10.1021/jacs.5c15469

**Published:** 2025-11-05

**Authors:** Yizhou Dai, Xinyue Zheng, Markus Antonietti, Mateusz Odziomek

**Affiliations:** Colloid Chemistry Department, 28321 Max Planck Institute of Colloids and Interfaces, Potsdam 14476, Germany

## Abstract

We report a nitrogen-doped carbon derived from tetracyanoquinodimethane (TCNQ900) that alone catalyzes nitrite reduction (NO_2_^–^ RR) to NH_3_ with an onset potential higher than +0.10 V vs RHE and catalytic activity rivaling state-of-the-art transition-metal catalysts. Contrary to the usual view that carbon supports merely modulate metal sites, we show the reverse: atomically dispersed Cu on TCNQ900 poisons active N-rich motifs, preventing the formation of a tandem catalyst in which NO_3_^–^ is first reduced to NO_2_^–^ by Cu and subsequently to NH_3_ by TCNQ900. Molecular analogues confirm that Cu coordination blocks N-rich pockets that drive the NO_2_^–^ RR. In contrast, physically mixing Cu nanoparticles with TCNQ900 restores a true tandem effect: Cu NPs reduce NO_3_^–^ to NO_2_^–^, while unpoisoned TCNQ900 converts NO_2_^–^ to NH_3_, achieving 4-fold higher partial currents. These findings overturn the “noninnocent” support concept and highlight the reciprocal influence of metals on carbon catalysts, providing new designing guidelines and pointing to the potential role of carbon supports as active actors in catalysis.

## Graphical abstract

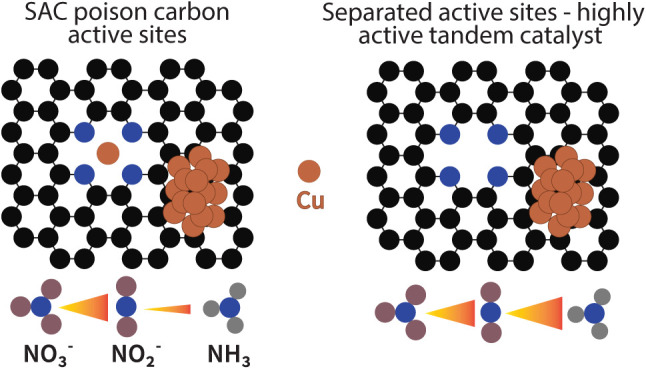

## 1 Introduction

Carbon materials are often simplified as inert supports for dispersing and stabilizing metal active sites, including in advanced metal–nitrogen–carbon (M–N–C) single-atom catalysts (SACs), where catalytic activity is typically attributed solely to the central metal atom.^1–4^ Heteroatom-doped carbons within M–N–C systems are usually regarded as “noninnocent substrates” that modulate the electronic structure of the metal center,^5–8^ with their direct catalytic role assumed negligible. While it is well established that metal–carbon support coordination can strongly influence metal activity,^2–9^ the reciprocal effect of metal incorporation on the catalytic behavior of the carbon support has received little attention.

Heteroatom-doped carbons have shown exceptionally high activity in oxygen reduction reaction (ORR)^10–12^ and in other electrocatalytic energy and chemical conversions,^13–16^ rivaling the best transition-metal catalysts. In this study, we demonstrate that a nitrogen-doped carbon (NDC) derived from tetracyanoquinodimethane (TCNQ), denoted here as TCNQ900,^17^ exhibits exceptional activity and selectivity for the electrochemical nitrite reduction reaction (NO_2_^–^ RR), with an onset potential as low as +0.1 V vs RHE. Its performance matches or even exceeds that of leading metal-based catalysts, and it remains highly active even at low NO_2_^–^ concentrations (10 mM), mimicking the behavior of heterogeneous nitroreductases.^18^ Such reaction is interesting not only for synthesis and waste management but also for redox-flow energy storage, as demonstrated by Jiang et al.^19^

In biological systems, the anaerobic nitrate reduction pathway is divided into two steps: nitrate-to-nitrite and nitrite-to-ammonia, both catalyzed by porphyrin-like structures with a central metal atom. A similar stepwise mechanism has been observed in heterogeneous electrocatalysis, described as the “2 + 6” electron pathway,^19–22^ as an alternative to the direct “8-electron” transfer route.^23^ Inspired by this model, we sought to exploit TCNQ900 as an efficient carbon support and cocatalyst for tandem nitrate reduction by coupling it with metallic species.

Contrary to expectations, introducing metal atoms in the form of SACs significantly suppressed the catalytic activity of TCNQ900. Rather than enhancing the reaction, the metal single atoms (Cu, Zn) acted as poisoning agents, blocking the highly active N-sites of the NDC. Using molecular analogues, we show that TCNQ900’s NO_2_^–^ RR activity stems from clustered nitrogen motifs that also serve as preferred anchoring sites for metal atoms. Upon coordination of single-atom Cu or Zn, these N-clusters become catalytically deactivated, providing direct evidence of the SAC-induced poisoning of active carbon centers. By physically mixing Cu nanoparticles with TCNQ900, thereby spatially decoupling metal and carbon domains, we fully restore the tandem NO_3_^–^ to NO_2_^–^ and NO_2_^–^ to NH_3_ pathway and achieve true cooperative catalysis. Our findings offer a new design paradigm for carbon-supported metal electrocatalysis: sequential, multistep reactions can mask the intrinsic role of high-surface-area carbons, and simple comparisons of loaded versus unloaded supports may vastly underestimate the carbon framework’s contribution to the overall activity.

## 2 Results

### 2.1 Performance of TCNQ900 in NO_2_^–^ RR and NO_3_^–^ RR

The NDC material was synthesized by thermal condensation of the TCNQ molecule in a ZnCl_2_:NaCl salt melt at 900 °C, following our previously reported procedure (Figure 1, see the methods in SI).^17^ Material characterization is described in Supplementary note 1 and presented in Figures S1–S5. The NO_3_^–^ RR and NO_2_^–^ RR performance of TCNQ900 was evaluated using an H-type electrochemical cell and 1 M NaOH as an electrolyte. Linear sweep voltammetry (LSV) revealed a significant increase in current density upon NaNO_2_ addition, which further increased with a higher concentration (Figure 2a). Notably, the onset potential for NO_2_^–^ reduction was observed at a potential higher than +0.1 V vs RHE, comparable to state-of-the-art transition-metal catalysts.^24–29^ The specific activity of N-heteroatoms in TCNQ900 was benchmarked against a commercially activated carbon (TF-B520) and an oxocarbon from our previous study (RC-SnK-800),^30^ both exhibiting comparable specific surface areas to TCNQ900 (Figure S6), with RC-SnK-800 having a comparable amount of heteroatom doping but with oxygen (Table S1). As shown in Figure 2b, both reference materials demonstrated negligible NO_2_^–^ RR activity, highlighting the special properties of N-heteroatoms in TCNQ900 in activating NO_2_^–^ ions.

**Figure 1:**
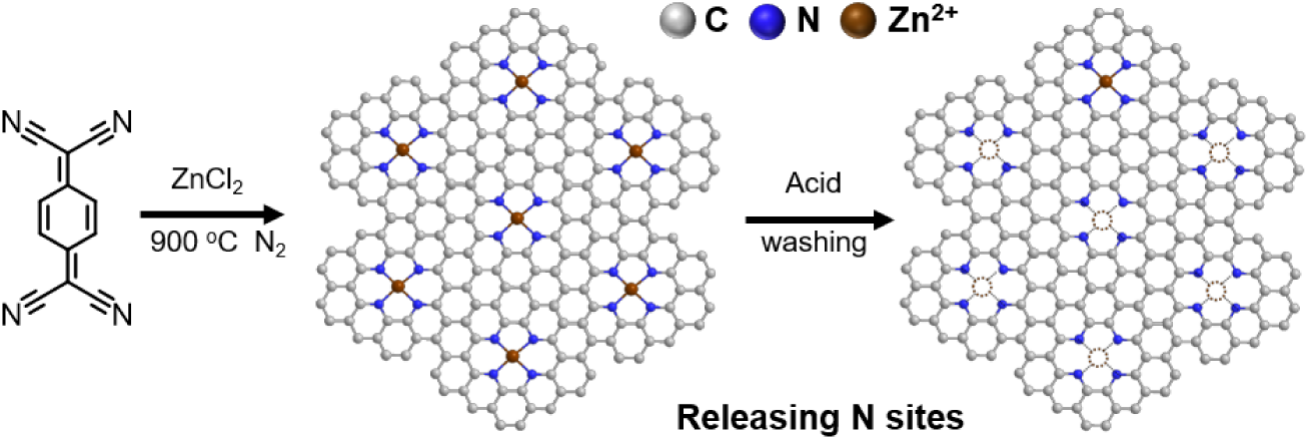
Schematics of the synthesis of TCNQ900.

**Figure 2:**
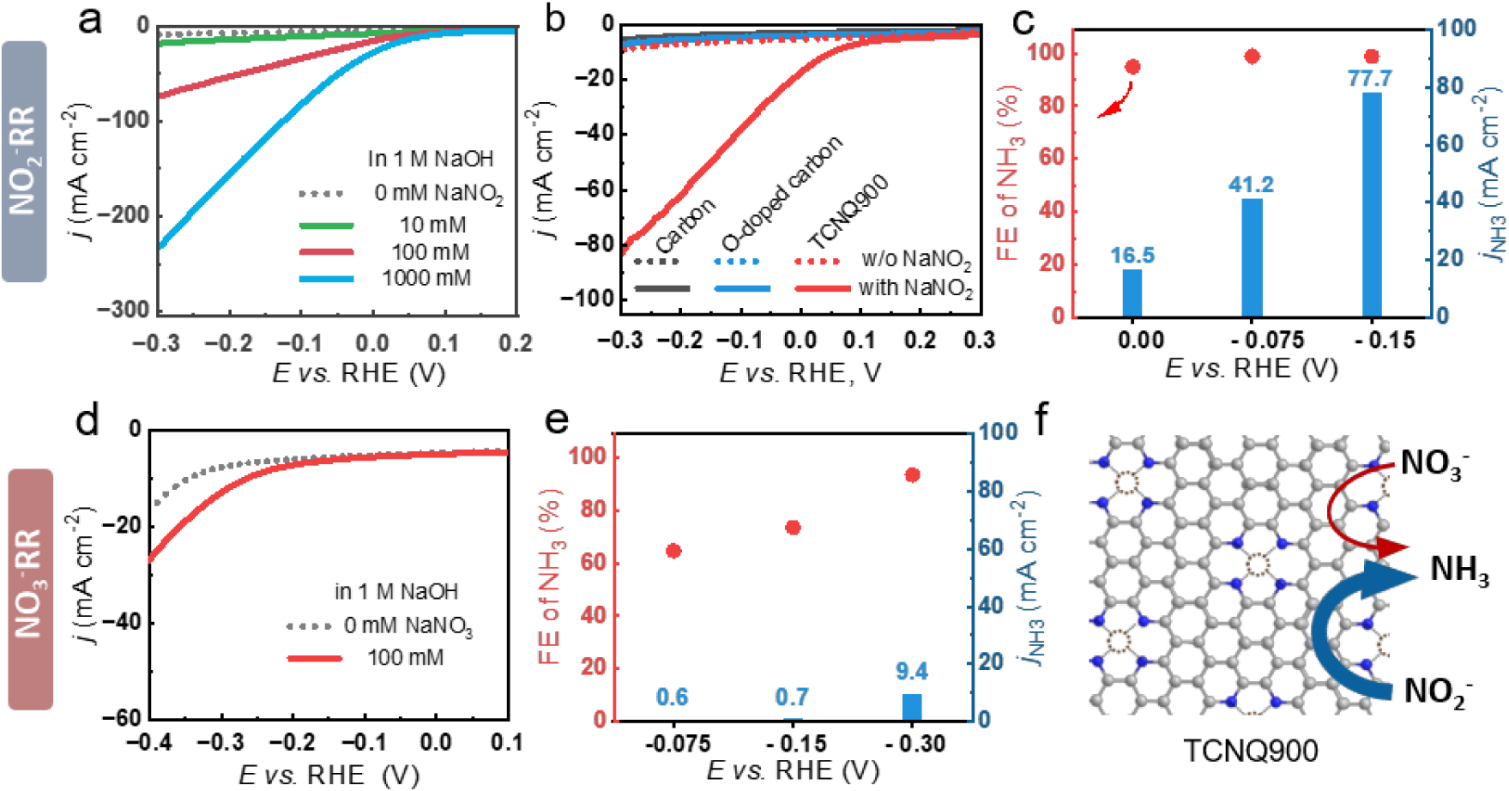
Electrochemical NO_*x*_^–^ reduction performance of TCNQ900. (a) LSV curves of TCNQ900 in 1 M NaOH with different concentrations of NaNO_2_. (b) LSV curves in 1 M NaOH + 100 mM NaNO_2_ comparing TCNQ900, carbon (TF-B520), and O-doped carbon (RC-SnK-800). (c) Bulk electrolysis performance of TCNQ900 on carbon paper in 1 M NaOH + 100 mM NaNO_2_ under different potentials for 0.5 h. (d) LSV curves of TCNQ900 in 1 M NaOH with and without 100 mM NaNO_3_. (e) Bulk electrolysis performance of TCNQ900 on carbon paper in 1 M NaOH + 100 mM NaNO_3_ under different potentials for 0.5 h. (f) Schematic illustration of the NO_2_^–^ RR and the NO_3_^–^ RR on TCNQ900; the thickness of arrows correlates to relative activities. For all LSV tests, the scan rate was 10 mV s^–1^, and the data were provided without *iR* compensation.

In the next step, bulk electrolysis was carried out with 100 mM NaNO_2_ at potentials between 0 and −0.15 V vs RHE for 0.5 h (Figure 2c) to accumulate and quantify emerging NH_3_. Increasing cathodic potentials resulted in higher partial current densities for NH_3_ production (*j*_NH3_), ranging from 16.5 mA cm^–2^ at 0 V to 77.7 mA cm^–2^ at −0.15 V vs RHE. Across the tested potential window, NH_3_ remained almost the only product, with faradaic efficiencies (FEs) exceeding 90% and reaching nearly 100% at −0.075 and −0.15 V vs RHE. Moreover, TCNQ900 showed excellent stability, maintaining its activity and selectivity over six consecutive runs at −0.15 V vs RHE (Figure S7). The yield rate of NH_3_ production from NO_2_^–^ at −0.15 V vs RHE was equal to 8.21 mg_NH3_ cm^–2^ h^–1^ and was competitive to the best transition-metal catalysts, far exceeding the reported carbon materials (Figure S8, Table S2).

In great contrast to NO_2_^–^ RR, TCNQ900 shows rather poor activity for NO_3_^–^ RR, as evidenced by a more negative onset potential of −0.10 V vs RHE and much lower current densities measured by LSV, reaching 27 mA cm^–2^ at −0.4 V in comparison to 18 mA cm^–2^ in the absence of nitrate (Figure 2d). Therefore, the performance in NO_3_^–^ reduction is strongly affected by competing HER. Further bulk electrolysis in 1 M NaOH using 100 mM NaNO_3_ also confirmed poor NH_3_ production (Figure 2e). Specifically, at the potential more negative than that of the tested bulk electrolysis for NO_2_^–^ RR (−0.30 V vs RHE), NO_3_^–^ RR reached an FE of 93.6% with a current density of 9.4 mA cm^–2^ for NH_3_ production, indicating the sluggish activation of NO_3_^–^ compared to that of NO_2_^–^. In the range of −0.075 to −0.15 V vs RHE, the activity is negligible.

TCNQ900’s exceptional NO_2_^–^ RR activity makes it an attractive partner for the NO_3_^–^ RR via the “2 + 6” electron pathway. In this mechanism, NO_3_^–^ is first reduced to NO_2_^–^, which desorbs and then readsorbs on an another catalyst or site for further reduction.^19–22^ By contrast, the direct “8-electron” route proceeds entirely at a single site, from NO_3_^–^ to NH_3_, without releasing an intermediate.^23^ Thus, when paired with an NO_3_^–^-activating catalyst, TCNQ900 would serve as an ideal cocatalyst in a tandem configuration. Given its high N-doping, hybridizing TCNQ900 with single-atom catalysts is a natural choice, and such catalysts have already proven effective in NO_3_^–^ RR.^31–36^ Recent experimental work, supported by DFT calculations, on 13 different M–N_*x*_ centers further suggests the prevalence of the “2 + 6” electron pathway in NO_3_^–^ to NH_3_ conversion on M–N–C materials.^21^ We therefore combine TCNQ900 with metallic species in the next section, aiming to realize such tandem catalysis.

### 2.2 Cu/TCNQ900-X% for NO_*x*_^–^ RR

Among the known candidates, copper stands out as one of the most effective metals for NO_3_^–^ RR, performing well both as SACs and nanoparticles (NPs).^37,38^ Notably, literature reports indicate that Cu exhibits relatively slow kinetics of NO_2_^–^ RR,^28,39^ suggesting complementary reactivity with TCNQ900. We employed a previously reported impregnation method^40^ to incorporate Cu species into TCNQ900 without altering its structural or chemical properties (see the methods part in SI). Two samples were synthesized with different nominal Cu loadings (1 and 11 wt %) to compare the performance of Cu SACs and NPs (Figure 3). These samples are denoted as Cu/TCNQ900-1% and Cu/TCNQ900-11%, respectively. As shown in Table S2, ICP-OES analysis determined the actual Cu contents of 0.8 and 5.7 wt % for Cu/TCNQ900-1% and Cu/TCNQ900-11%, respectively. Additional characterizations were performed to elucidate the state of the incorporated Cu species (Figures S9–S12, Table S1). XRD analysis revealed no detectable Cu-related reflections in Cu/TCNQ900-1%, while Cu/TCNQ900-11% displayed distinct peaks corresponding to metallic Cu (Figure S9). Elemental analysis confirmed consistent C:N ratios across Cu-loaded and -not-loaded samples (Table S1). HR-TEM imaging showed no noticeable changes in the carbon matrix after Cu incorporation (Figure S10 and Figure S11). Furthermore, TEM-EDX elemental mapping indicated the uniform distribution of all elements in both Cu-loaded samples (Figure S10 and Figure S11). Despite the presence of XRD-visible metallic Cu in Cu/TCNQ900-11%, the elemental distribution remained homogeneous, suggesting the formation of metallic Cu NPs or clusters smaller than a few nanometers and the coexistence of SACs. High-resolution HAADF-STEM images provided direct evidence of atomically dispersed Cu species in Cu/TCNQ900-1%, and of both atomically dispersed Cu and small metallic Cu clusters in Cu/TCNQ900-11% (Figure S12). A schematic representation of the evolving active site distribution with increasing Cu content is shown in Figure 3a.

**Figure 3:**
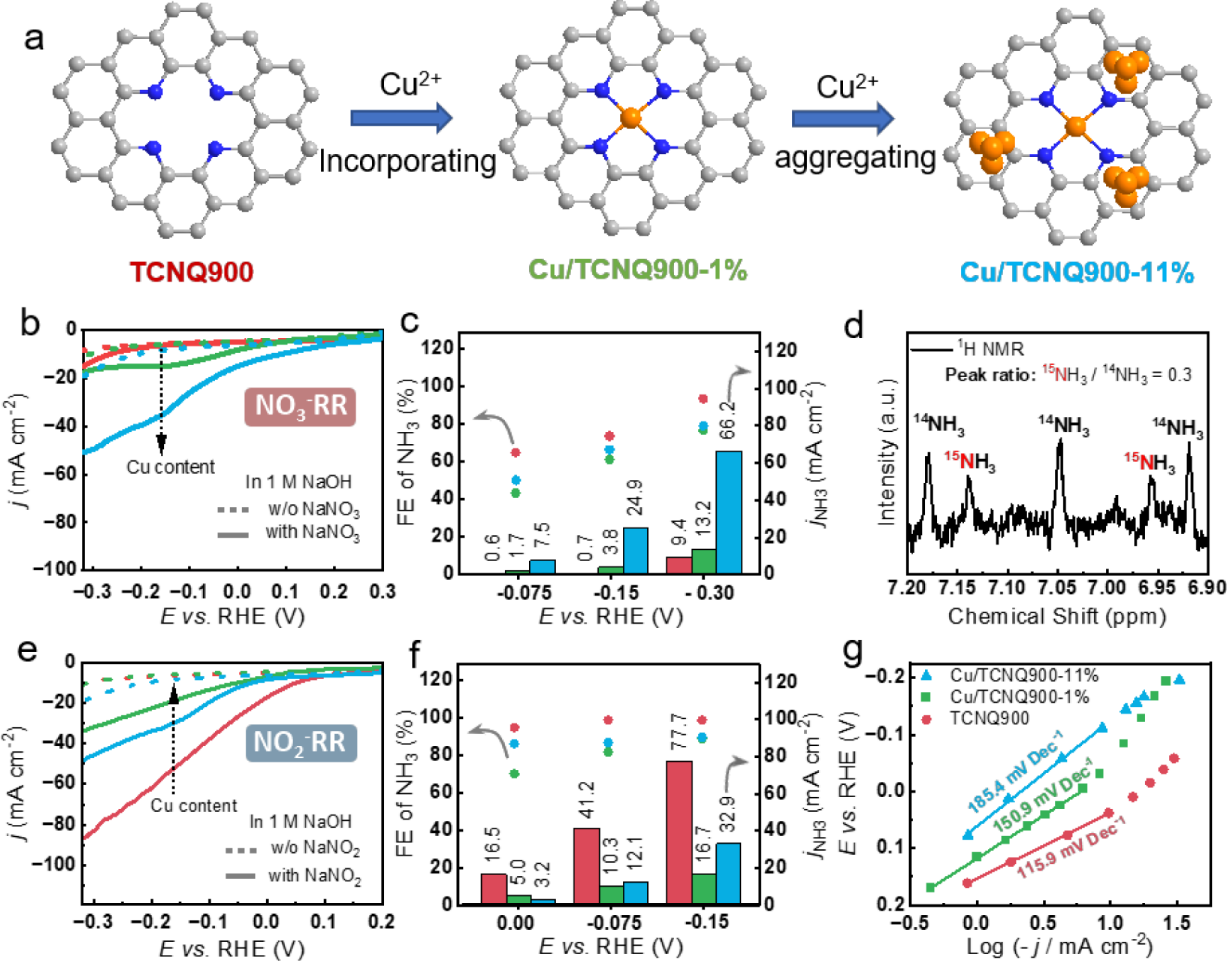
Electrochemical NO_*x*_^–^ reduction performance of Cu/TCNQ900-x%. (a) Schematic picture of active site variation within TCNQ900, Cu/TCNQ900-1%, and Cu/TCNQ900-11%, in the following graphs, the samples are represented by red, green, and blue color, respectively. (b) LSV curves of TCNQ900, Cu/TCNQ900-1%, and Cu/TCNQ900-11% in 1 M NaOH with and without 100 mM NaNO_3_, 10 mV s^–1^, and without *iR* compensation. (c) Bulk electrolysis performance of TCNQ900, Cu/TCNQ900-1%, and Cu/TCNQ900-11% in 1 M NaOH + 100 mM NaNO_3_ under different potentials for 0.5 h. (d) ^1^H NMR spectrum of the electrolyte after electrolysis with Cu/TCNQ900-1% in 1 M NaOH with 0.5 mM ^15^NO_2_^–^ and 100 mM ^14^NO_3_^–^. (e) LSV curves of TCNQ900, Cu/TCNQ900-1%, and Cu/TCNQ900-11% in 1 M NaOH with and without 100 mM NaNO_2_, 10 mV s^–1^, and without *iR* compensation. (f) Bulk electrolysis performance of TCNQ900, Cu/TCNQ900-1%, and Cu/TCNQ900-11% in 1 M NaOH + 100 mM NaNO_2_ under different potentials for 0.5 h. (g) Comparison of Tafel slopes of TCNQ900, Cu/TCNQ900-1%, and Cu/TCNQ900-11% in 1 M NaOH + 100 mM NaNO_2_.

### 2.3 Performance of Cu/TCNQ900-X% in NO_3_^–^ RR

The NO_3_^–^ RR activity and selectivity of Cu-loaded TCNQ900 were benchmarked against those of pristine TCNQ900. LSV in 1 M NaOH with 100 mM NaNO_3_ (Figure 3b) shows that increasing Cu loading raises current density and lowers onset potential, confirming Cu’s role in enhancing NO_3_^–^ RR. Nevertheless, the Cu/TCNQ900-1% sample exhibits low currents with a diffusion-limited plateau (to be explained further in the text). In contrast, Cu/TCNQ900-11% showed much more positive onset potential and higher, nonlimited currents, in compared to the other two samples. Bulk electrolysis in the potential range between −0.075 to −0.30 V vs RHE (Figure 3c) revealed that pristine TCNQ900 gives higher FE than loaded samples reaching over 95% at −0.3 V vs RHE, while all Cu-loaded samples remain below 80%. Moreover, Cu/TCNQ900-1% offers only marginal partial current density enhancement (1.7–13.2 mA cm^–2^) over the unloaded support (0.6–9.4 mA cm^–2^). In contrast, Cu/TCNQ900-11% delivers up to *j*_NH3_ of 70 mA cm^–2^ at −0.30 V vs RHE.

Depositing Cu NPs markedly enhances the NO_3_^–^ RR activity, whereas atomically dispersed Cu yields NH_3_ production comparable to that of pristine TCNQ900. Despite its lower overall NH_3_ yield, TCNQ900 alone delivers a higher FE at every tested potential. The reduced FE of the Cu-loaded samples does not stem from side reactions but rather from the accumulation of the NO_2_^–^ intermediate. Indeed, analytical quantification (Figure S13) confirmed the concurrent buildup of NH_3_ and NO_2_^–^ during NO_3_^–^ RR on both Cu/TCNQ900-1% and Cu/TCNQ900-11%. The persistent NO_2_^–^ accumulation is rather unexpected given TCNQ900’s high NO_2_^–^ RR activity. The increasing nitrite concentration may explain the diffusion-limited currents observed for Cu/TCNQ900-1%, suggesting that under these conditions, NO_2_^–^ conversion becomes rate-limiting.

Despite the matching potential windows between NO_3_^–^ RR on Cu/TCNQ900-X% and NO_2_^–^ RR on TCNQ900, the results point to very low tandem effect between Cu and TCNQ900 active sites in NO_3_^–^ RR. First speculation would be that in loaded samples direct “8-electron” reduction of NO_3_^–^ dominates NH_3_ production, i.e., the production of NH_3_ from NO_3_^–^ does not rely on desorbed NO_2_^–^. In such a case, the tandem effect between both components cannot be set in. To verify this speculation, we tested the competition between NO_3_^–^ and NO_2_^–^ reduction to ammonia. For that we performed electrochemical NO_*x*_^–^ reduction in a mixture of labeled ^15^NO_2_^–^ and standard ^14^NO_3_^–^ as elucidated in previous work.^21^ In this case, by simply using ^1^H NMR, we can distinguish ^14^NH_3_ and ^15^NH_3_ produced from nitrate and nitrite, respectively. The experiment was first carried out on Cu/TCNQ900-11%. The mixed electrolyte was composed of 2.5 mM ^15^NO_2_^–^ vs 100 mM ^14^NO_3_^–^, based on [NO_2_^–^]-time profiles in Figure S13, which showed nearly 3 mM of NO_2_^–^ accumulated during the first 0.5 h of electrolysis. Under a constant polarization of −0.15 V vs RHE, we passed 90 C of charge (≈0.93 mmol e^–^, which is roughly two times of the charge needed to fully convert the included concentration of ^15^NO_2_^–^ to ^15^NH_3_) and periodically sampled the electrolyte for ^1^H NMR analysis (Figure S14). At each sampled point, the ^1^H NMR spectrum exhibited negligible peaks from^15^NH_3_. Moreover, the ^15^NH_3_/^14^NH_3_ ratio was lower or just comparable to the initial ratio of ^15^NO_2_^–^/^14^NO_3_^–^, indicating that NO_2_^–^ to NH_3_ does not show superiority to NO_3_^–^ to NH_3_ on Cu/TCNQ900-11%. Considering that it still needs around 0.5 h to accumulate a similar concentration of NO_2_^–^ as in the doping experiment, NH_3_ production on this electrocatalyst seems to be less dependent on the desorbed NO_2_^–^ intermediate.

However, when it comes to Cu/TCNQ900-1%, the response was much different. Similarly, a mixed electrolyte of 0.5 mM ^15^NO_2_^–^ vs 100 mM ^14^NO_3_^–^ was used based on [NO_2_^–^]-time profiles shown in Figure S13. Figure 3d shows the ^1^H NMR spectra for such labeled experiment, after passing ∼30 C e^–^, which corresponds to about 0.31 mmol of e^–^ (roughly 3.5 times of the charge needed to fully convert the used concentration of ^15^NO_2_^–^ to ^15^NH_3_). Even at such a low concentration ratio of ^15^NO_2_^–^ vs ^14^NO_3_^–^, the ratio of produced ^15^NH_3_ and ^14^NH_3_ was comparable. Specifically, the peak ratio of as-produced ^15^NH_3_ to ^14^NH_3_ is 0.30, almost 60 times of initial [^15^NO_2_^–^]/[^14^NO_3_^–^], demonstrating that NO_2_^–^ is more effectively converted to NH_3_ than NO_3_^–^ on Cu/TCNQ900-1%. Considering the accumulation of nitrite during NO_3_^–^ RR and strong contribution of NO_2_^–^ in NH_3_ formation, the “2 + 6” electron pathway plays a main role in NO_3_^–^ to NH_3_ conversion on Cu/TCNQ900-1% in accordance with the previous report.^21^ Taking into account that ^14^NH_3_ produced via the ^14^NO_3_^–^ to ^14^NO_2_^–^ to ^14^NH_3_ route also takes place, the actual contribution of the “2 + 6” e^–^ pathway is likely even larger. Given the concomitant NO_2_^–^ accumulation, we investigate the reason for the massively lowered NO_2_^–^ RR activity of Cu/TCNQ900-1% in the next section.

### 2.4 Performance of Cu/TCNQ900 in NO_2_^–^ RR

We assessed the NO_2_^–^ RR performance of the Cu-loaded samples and compared it to that of pristine TCNQ900. To our surprise, LSVs show that even 1 wt % Cu input sharply decreases NO_2_^–^ RR: the onset potential shifts toward more negative potentials, and the current density at −0.30 V vs RHE falls from −90 to −35 mA cm^–2^ (Figure 3e). Raising the Cu loading to 11 wt % partially restores activity, reaching −50 mA cm^–2^ at −0.30 V, but still underperforms unloaded TCNQ900. This partial performance recovery is likely due to the contribution of Cu NPs, which are known to exhibit some NO_2_^–^ RR activity.^29^

In bulk electrolysis, both the partial current density for NH_3_ (*j*_NH3_) and the FE decreased significantly upon Cu loading (Figure 3f). The unloaded sample showed 4 to 5 times higher activity and almost quantitative formation of NH_3_ in comparison to 70–90% FE for Cu-loaded samples. Tafel slopes further corroborate kinetic deterioration, increasing from 116 mV dec^–1^ (TCNQ900) to 151 and 185 mV dec^–1^ for Cu/TCNQ900-1% and Cu/TCNQ900-11%, respectively (Figure 3g). We also ruled out support degradation by preparing a control sample (Cu/TCNQ900-0%) under identical conditions but without Cu salts. Structural and chemical analyses (Figures S15–S19, Table S1) and NO_2_^–^ RR measurements (Figure S20) were indistinguishable from pristine TCNQ900, confirming that the activity decline stems solely from Cu incorporation.

The results clearly demonstrate that atomically dispersed Cu on TCNQ900 severely inhibits the NO_2_^–^ RR by poisoning the most active N sites. As Cu loading increases, clusters or NPs introduce alternative NO_2_^–^ reduction sites, but coexisting single-atom sites continue to block the much more active N-doped carbon motifs. Since NO_3_^–^ RR on Cu/TCNQ900-11% proceeds mainly via the 8-electron pathway, and TCNQ900 is unable to engage in the parallel “2 + 6” e^–^ route, the carbon matrix present there serves merely as a rather inert support for Cu NPs. The effect is particularly detrimental in Cu/TCNQ900-1%, which relies largely on the “2 + 6” e^–^ pathway. As a result, the intended tandem function is disrupted. Cu still reduces NO_3_^–^ to NO_2_^–^, but the subsequent NO_2_^–^ to NH_3_ step is inefficient on the Cu single-atom site. These findings indicate that the most catalytically active N-sites also serve as preferred anchoring points for Cu, likely corresponding to clustered nitrogen motifs. In the following section, we examined the chemical identity and structure of these motifs in greater detail.

### 2.5 Origin of NO_2_^–^ RR Performance and Cu-Induced Deactivation Mechanism on TCNQ900

A major challenge in carbon-based catalysis lies in identifying the true nature of active sites, as heteroatoms such as nitrogen can occupy a variety of positions within the carbon lattice, resulting in diverse local electronic environments. Even in extensively studied reactions like the ORR, there remains no consensus on the precise nature of the active sites in NDCs.^11,41^ This complexity is evident in XPS measurements, where nitrogen typically gives rise to at least four distinct components—pyridinic, pyrrolic, graphitic, and oxidized nitrogen—within a relatively broad 4 eV binding energy range, and this is the same for TCNQ900 (Figure S4). Moreover, clustering of nitrogen atoms, as observed in M–N–C catalysts, further alters the coordination environment and activity/selectivity in catalytic reactions. For instance, one pyrrolic N might not be very active, but the porphyrin structure with cavity made by four pyrrolic N could serve as an active center.^42^

The thermal condensation of TCNQ in a ZnCl_2_-based salt melt yields the present covalent carbon network with nitrogen atoms embedded in the framework.^17^ In this synthesis, Zn^2+^ plays a key role by coordinating with N atoms from TCNQ and forming N-rich “pockets” capable of stabilizing cations.^43^ These Zn-containing sites are liberated by acidic washing, creating vacant coordination environments that may serve as anchoring sites for other metal cations such as Cu (Figure 1). This observation led us to hypothesize that the active sites responsible for the NO_2_^–^ RR are precisely those Zn-coordinated N-pockets, liberated upon Zn removal.

To test this hypothesis and to prove that residual Zn cations do not participate in NO_*x*_^–^ RR, we prepared a series of TCNQ900 samples with varying Zn contents by modifying the strength of postsynthesis washing conditions: washing with deionized water (TCNQ900-H_2_O), 1 M HCl (TCNQ900), and 5 M HCl (TCNQ900-5 M HCl). The structural, chemical, and morphological characterization revealed negligible differences among the three materials (Figures S21–S25). ICP-OES analysis confirmed progressive Zn removal with increasing acid strength, yielding residual Zn contents of 2.17, 0.76, and 0.44 wt %, respectively (Table S3), as schematically shown in Figure 4a. We further observed the variation of N species upon Zn removal from XPS N 1*s* spectra (Figure S26), confirming that the removal of Zn liberates a certain type of N (Pyrrolic N) (Figure S27) and showing good alignment with other reported NDCs involving a similar Zn^2+^-assisted salt-templating method.^43^

**Figure 4:**
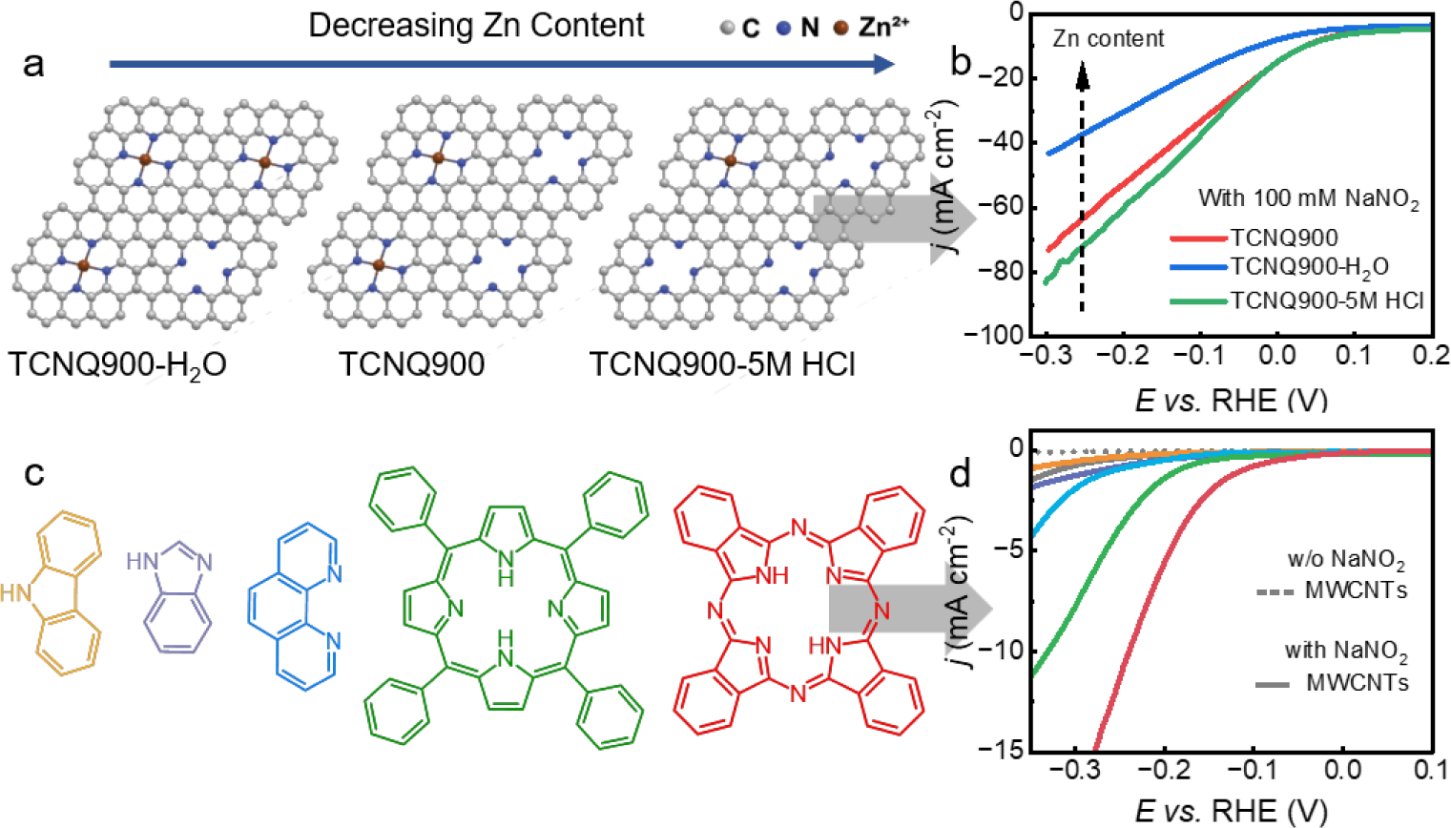
Investigation of the origin of NO_2_^–^ RR performance on TCNQ900. (a) Schematic illustration of N_*x*_ sites and (b) comparison of LSV curves in 1 M NaOH + 100 mM NaNO_2_ of TCNQ900 samples with different contents of residual Zn cations. (c) Structural formula of conjugated molecules mimicking the active sites of TCNQ900, from left to right in order: Carbazole, Benzimidazole, 1,10-Phenanthroline, 5,10,15,20-Tetraphenyl-21H,23H-porphine, 29H,31H-Phthalocyanine, and the corresponding (d) LSV curves in NO_2_^–^ RR of model catalysts dispersed on carbon nanotubes, with different colors representing each molecule; the scan rate was 10 mV s^–1^, and all of the electrochemical results were provided without *iR* compensation.

According to our hypothesis, LSV measurements with 100 mM NaNO_2_ confirmed a significant increase in current density with decreasing Zn content (Figure 4b), while onset potentials remained largely unchanged. Such difference in current density became more obvious at higher NO_2_^–^ concentration (1000 mM), while all samples exhibited overlapping LSV curves at lower NO_2_^–^ concentration (10 mM) (Figure S28), further suggesting that the enhanced current arises from an increased number of accessible active sites. Therefore, the active sites for the NO_2_^–^ RR are associated with clustered N atoms, forming N-rich cavities, released upon Zn removal.

Given the harsh thermal treatment involved in TCNQ900 synthesis, we have to assume that multiple types of N-environments and cavities coexist. This makes the precise determination of active sites difficult. For that reason, we used an indirect method to study the possible active sites by using model molecular systems mimicking the active sites. Prior studies have shown that heteroatom-doped, π-conjugated organic molecules can simulate specific N environments in carbon materials^44,45^ and also can be anchored by π–π interaction onto conductive substrates. We therefore selected a set of well-defined conjugated molecules: carbazole, benzimidazole, 1,10-phenanthroline, porphyrin, and phthalocyanine, having different amounts of N atoms and representing different arrangement types of N-doping atoms (Figure 4c). These molecules were immobilized on multiwalled carbon nanotubes (MWCNTs) via established π–π interaction methods.^46^ The electrochemical activity of such prepared materials has been further tested in the NO_2_^–^ RR.

LSV measurements confirmed that pristine MWCNTs are largely inactive toward the NO_2_^–^ RR, offering a clean baseline for evaluating molecular catalysts. A clear trend emerged among the model compounds: molecules with higher nitrogen content and clustered N atoms exhibited significantly enhanced NO_2_^–^ RR activity, with lower onset potentials and higher current densities (Figures 4d and S29). The normalized activities to the overall nitrogen loading did not change this trend (Figure S30). More specifically, phenthalocyanine (Pc), with its well-defined N_4_/N_8_ macrocyclic cavity, showed by far the highest activity. In contrast, carbazole, containing a single pyrrolic N atom, was nearly inactive, despite sharing the same nitrogen type as Pc, highlighting the importance of multiple N-sites. These results challenge the prevailing single-heteroatom view of active sites in N-doped carbons (e.g., pyridinic, pyrrolic, or graphitic nitrogen atoms). It is worth noting that TCNQ900 exhibits significantly higher activity than even the most active Pc, owing to the embedding of N-rich cavities within a conductive carbon network. This extended π-conjugated system modifies the electronic environment of the active sites, thereby enhancing catalytic performance. Such electronic band engineering offers a distinct advantage over the discrete and finite electronic states of molecular catalysts.

In fact, Zn–N_*x*_ motifs in NDCs synthesized via ZnCl_2_ melts are frequently represented as Pc-like structures.^43^ Building on this analogy, we next examined how metal coordination affects the NO_2_^–^ RR performance of Pc, which serves as a “molecular model” of the active site. This was readily achieved by using commercially available Cu-coordinated phthalocyanine (CuPc). Both CuPc and Pc were immobilized on MWCNTs and tested electrochemically. As expected, CuPc/MWCNTs showed a more negative onset potential and reduced NO_2_^–^ RR activity in comparison to Pc/MWCNTs (Figures 5a, S31), consistent with the suppression observed in Cu/TCNQ900-X%. In contrast, under NO_3_^–^ RR conditions (1 M NaOH, 100 mM NaNO_3_), CuPc/MWCNTs outperformed Pc/MWCNTs, with lower onset potential and higher current density (Figures 5b, S32), again paralleling the trends in Cu/TCNQ900.

**Figure 5:**
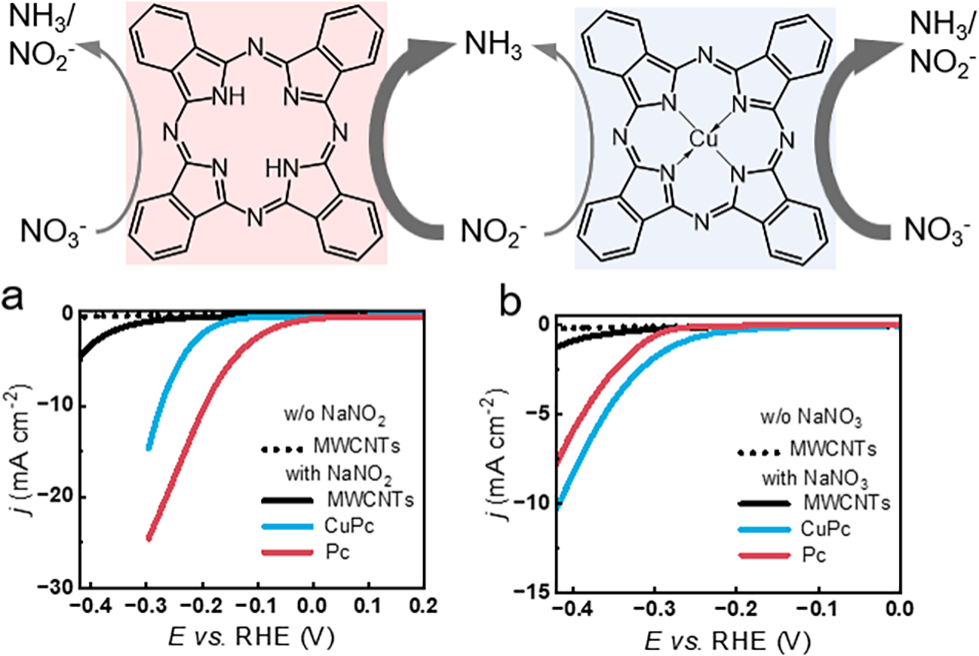
Investigation of the model 29H,31H-Phthalocyanine molecule with and without coordinated Cu cations. (a) Comparison of LSV curves in the NO_2_^–^ RR between Pc/MWCNTs and CuPc/MWCNTs. (b) Comparison of LSV curves in NO_3_^–^ RR between Pc/MWCNTs and CuPc/MWCNTs. The scan rate was 10 mV s^–1^, and all of the electrochemical results were provided without *iR* compensation.

### 2.6 Optimized Design of Cu-TCNQ Tandem Catalyst

All of these insights guided the design of a tandem catalyst in which commercial Cu NPs are physically mixed with TCNQ900, thereby avoiding direct coordination of Cu atoms to active N-sites (Figure 6a). XRD and TEM analysis were performed on such commercial Cu NPs, as shown in Figures S33 and S34. Here, we can test whether spatial decoupling could restore the NO_3_^–^ to NO_2_^–^ and NO_2_^–^ to the NH_3_ cascade. Remarkably, this physically mixed (PM) sample delivered much higher NO_3_^–^ RR current densities than bare Cu NPs even at mild overpotentials (Figure 6b) and exhibited both greater NH_3_ partial current densities and FE under all tested conditions (Figure 6c). The sluggish NO_2_^–^ RR of unsupported Cu NPs leads to accumulation of NO_2_^–^ instead of NH_3_ and thus only 20–40% FE for ammonia after 0.5 h of electrolysis across the selected potential window. In contrast, the PM configuration promotes concomitant efficient NO_2_^–^ RR boosting FE above 90% and increasing current density 4-fold. Control LSVs in NO_2_^–^ RR confirm that the PM sample’s performance mirrors that of pristine TCNQ900 (Figure S35), while Cu NPs alone remain poorly active for NO_2_^–^ RR (Figure S36), demonstrating that the observed enhancement indeed arises from a true tandem effect. Furthermore, at comparable Cu loadings, the PM catalyst far outperforms Cu/TCNQ900-11%, achieving 95.0 mA cm^–2^ at −0.14 V vs RHE versus only 24.9 mA cm^–2^ at −0.15 V vs RHE for the anchored material, underscoring the critical role of preserving TCNQ900’s NO_2_^–^ RR activity while harnessing Cu’s NO_3_^–^ RR capability.

**Figure 6:**
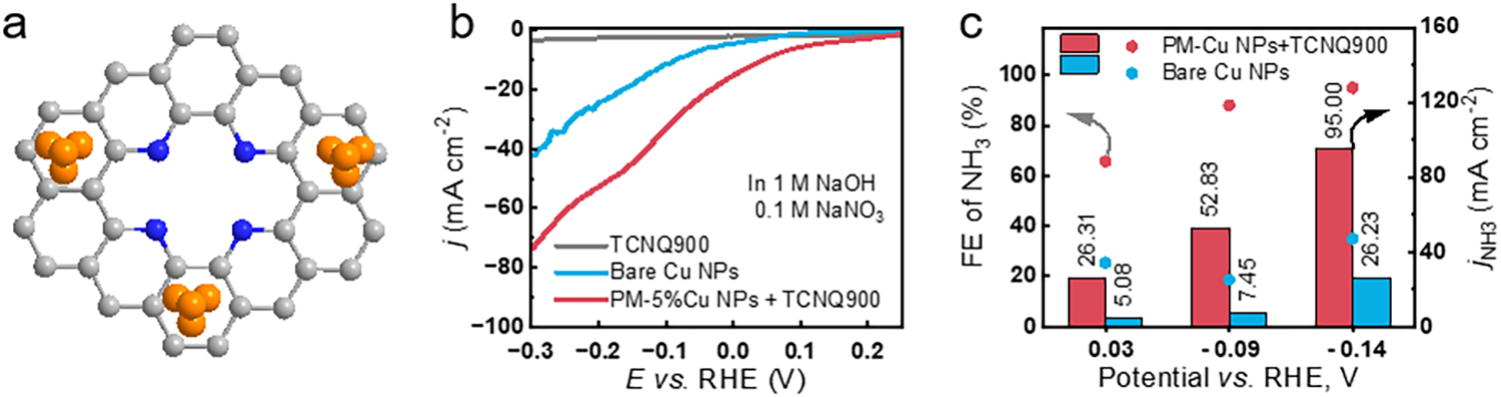
Optimizing the architecture of tandem catalysts: the synergy between Cu sites and N-sites in TCNQ900 by physical mixing. (a) Schematic illustration of active sites in PM-5% Cu NPs + TCNQ900. (b) LSV curves in 1 M NaOH + 100 mM NaNO_3_ of TCNQ900, bare Cu NPs, and physically mixed Cu NPs with TCNQ900 (denoted as PM-5% Cu NPs + TCNQ900). (c) Comparison of the bulk electrolysis performance in the NO_3_^–^ RR between bare Cu NPs and PM-5% Cu NPs + TCNQ900 under different potentials for 0.5 h.

## 3 Conclusions

This work demonstrates that efficient 6-electron electroreduction of nitrite to ammonia can be achieved using a nitrogen-doped carbon (TCNQ900), with performance matching or exceeding that of transition-metal catalysts. However, a straightforward extension of this system into a tandem “2 + 6” electron nitrate reduction pathway, by incorporating atomically dispersed Cu, unexpectedly failed. Instead of enhancing the performance, single-atom Cu and Zn strongly suppressed the NO_2_^–^ RR activity of the carbon support.

Model experiments using molecular analogues reveal that the high activity of TCNQ900 originates from clustered nitrogen motifs, which also act as preferential anchoring sites for metal atoms. Upon metal coordination, these sites become catalytically deactivated, providing direct evidence that single-atom metals can be poisoning agents in certain tandem architectures.

Learning from these insights, we revised a tandem design by spatially separating Cu nanoparticles and N-rich carbon sites. This configuration successfully realizes the targeted “2 + 6” electron nitrate-to-ammonia conversion, where carbocatalysis (NO_2_^–^ RR) and Cu nanoparticle catalysis (NO_3_^–^ RR) work in concert. These findings establish a new blueprint for designing cooperative catalytic systems that harness the strengths of both metal and metal-free components, paving the way for rethinking carbon’s role in multielectron electrocatalysis.

## Supplementary Material



## Notes

Synthetic protocols, characterization methods, and protocols of electrochemical analyses; Supplementary Note 1: characterization of TCNQ900; supplementary figures including XRD, elemental analysis, XPS, N_2_ sorption at 77 K, TEM, SEM, electrochemical stability of TCNQ900 in NO_2_^–^ RR, ^1^H NMR spectra, and additional electrochemical data (PDF)
