# Supplementary material for: Nitrite Reduction at Low Overpotentials on N‑Doped Carbon: When Metal Single Atoms Become Poisons
Source: J Am Chem Soc. 2025 Nov 5;147(46):42874–82. doi: 10.1021/jacs.5c15469 (PMC12636005; doi:10.1021/jacs.5c15469)
Supplement: Supplementary file 1 [file ja5c15469_si_001.pdf]

Supporting Information

Nitrite Reduction at Low Overpotentials on N-Doped Carbon: When Metal Single Atoms Become Poisons

Yizhou Dai,[1] Xinyue Zheng,[1] Markus Antonietti,[1] Mateusz Odziomek[1*]

[1] Colloid Chemistry Department, Max Planck Institute of Colloids and Interfaces, 14476 Potsdam, Germany

Corresponding author email: mateusz.odziomek@mpikg.mpg.de

Methods

Chemicals:

7,7,8,8-Tetracyanoquinodimethane (TCNQ, >98.0%), Copper (II) phthalocyanine (CuPc, □-form, >98%) were purchased from TCI chemicals. Ethanol, Zinc chloride anhydrous ($ZnCl_2$, >98%), sodium chloride (NaCl, >99.5%), and hydrochloric acid (~37%) were obtained from Thermo Fisher. Deionized water was prepared using a Merck Milli-Q system (25 MΩ/cm). TF-B520 activated carbon was purchased from MTI Corporation All the chemicals were used without any further purification. Carbon paper was ordered from Fuel Cell Store. Copper chloride ($CuCl_2$), (DMF), Multiwall carbon nanotubes (MWCNTs) with an inner diameter of 6-13 nm, Carbozole (≥95%), Benzimidazole (>98%), 1,10-Phenanthroline (≥99%), 5,10,15,20-Tetraphenyl-21*H*,23*H*-porphine (≥99%), 29H,31H-Phthalocyanine (□-form, 98%), Poly (vinylidene fluoride) (PVDF, average M.W. ~ 534,000), Sodium Nitrate ($NaNO_3$, ≥99.0%), Sodium Nitrite ($NaNO_2$, ≥99.0%), Commercial Copper nanoparticles (Cu NPs, 40-60 nm particle size (SAXS), ≥99.5% trace metals basis, reference number 774111) were purchased from Merck. Sodium Nitrate – ^{15}N ($Na^{15}NO_2$, ≥99.0% of atom %, ≥98.5%) was purchased from Aladdin.

Synthesis of TCNQ900:

The synthesis of TCNQ900 was following our previous work[1]. Specifically, one gram of TCNQ (1.0 g) was finely ground in a mortar to achieve a very fine powder. Subsequently, 5 g each of $ZnCl_2$ and NaCl were added, and the grinding process continued to ensure thorough mixing. The resulting powder was transferred to a crucible and flushed with nitrogen gas for one h before being heated. The heating protocol involved an initial ramp to 300 °C at a rate of 120 °C h^{-1}, with the temperature held steady at 300 °C for 1 h. This was followed by calcination at 900 °C, with a slower heating rate of 60 °C h^{-1} and a prolonged duration of 3 h at the target temperature. After calcination, the samples were washed twice with 500 mL of 1 M HCl and once with deionized water, for 24 h each step. The final material was vacuum-dried overnight at 150 °C, stored in air, and denoted as TCNQ900. Other samples with different contents of Zn residue were prepared by substituting1 M HCl with DI-water and 5 M HCl, denoted as TCNQ900-H_2O and TCNQ900-5 M HCl, respectively.

Synthesis of Cu/TCNQ900:

The synthesis of Cu/TCNQ900 was following a reported work[2] with the two-step annealing method. Specifically, certain amount of $CuCl_2$ and TCNQ900 (100 mg) were dispersed in ethanol solution (20 ml) and sonicated for 10 min, followed by rotary evaporation and then in an oven at 80 °C. For low-temperature annealing, the powder was heated to 300 °C (heating rate, 5 °C min^{-1}) for 5 h in a nitrogen flow. After thorough washing using a water–ethanol mixture, the dried powders (80 °C) were subjected to a high-temperature annealing at 550 °C (heating rate, 2 °C min^{-1}) for 5 h in a nitrogen flow. Considering the input mass ratio of Cu, two samples were denoted as Cu/TCNQ900-1% and Cu/TCNQ900-11%, respectively.

Synthesis of model catalysts with conjugated organic molecules loaded on MWCNTs:

The loading of a series of N-containing molecules on MWCNTs was achieved through a π-π stacking method reported previously.[3] We first calcined as-received MWCNTs at 500 °C in air for 1 h. The calcined MWCNTs were sonicated in a 5 wt.% HCl aqueous solution for 30 min,

followed by stirring overnight. Then, the MWCNTs were washed with deionized water until pH neutral and collected by centrifuging. Taking the loading of carbazole on MWCNTs as an example, 10.0 mg of purified MWCNTs in 10 ml DMF and 1 mg carbazole in 10 ml DMF were separately prepared, and each was sonicated for 1 h. Then, the two solutions were merged and sonicated for another 1 h followed by stirring at room temperature for 20 h. Subsequently, the solution was centrifuged, and the supernatant was discarded. The precipitate was washed with DMF and centrifuged several times. Finally, the product was washed with deionized water and centrifuged twice before it was freeze dried to yield Carbazole/MWCNTs. For the loading of other molecules, the input mass ratio of each molecules was also maintained as 10%. Thusly, Benzimidazole/MWCNTs, Phenanthroline/MWCNTs, Porphrin/MWCNTs, Pc/MWCNTs, and CuPc/MWCNTs were also prepared.

Characterizations:

Powder XRD was performed on a Smartlab Studio II powder X-ray diffractometer with Cu Kα irradiation (λ=1.54 Å). FTIR spectroscopy was carried out using a Thermo Scientific Nicolet iD5 spectrometer with an attenuated total reflection (ATR) sampling technique. XPS measurements were performed on a Thermo Scientific K-Alpha. ThermoScientific Avantage software was used to analyze the resulting spectra. The binding energy was calibrated against the 284.8 eV peak of carbon (C–C). TGA was performed using a NETZSCHTG 209 F1 device in the range of 25 °C to 1000 °C in a nitrogen and air atmosphere with a heating rate of 10 °C min^{-1} in a Pt crucible. TGA-MS was carried out from 25 °C to 900 °C in a helium atmosphere with a heating rate of 2 °C min^{-1} in a Pt crucible. The physisorption analysis was done by N_2 sorption at 77 K on the Quantachrome Quadrasorb SI apparatus. Before the measurement, all the samples were degassed at 150 °C for 20 h under a vacuum system. Specific surface areas (SSA) were calculated from the N_2 adsorption data at the p/p_0 range from 0.05 to 0.25 by the multipoint BET model. Scanning Electron Microscopy (SEM) was performed on LEO 1550 Gemini Zeiss microscope at 5 keV beam energy after sputtering of 5 nm of gold on top of the samples. EDX was performed using an Oxford Instruments EDX analyzer. Scanning transmission electron microscopy (STEM) was performed using a double-Cs corrected JEOL JEM-ARM200F, operated at 80 kV using a probe semi-convergence angle of 23 mrad, equipped with a cold-field emission gun. ICP measurements were done on the PerkinElmer ICP-OES Optima 8000. For that, 5-10 mg of material was dissolved in a mixture of 500 µL HNO3 and 1500 µL HCl overnight to determine Zn and Cu contents. After digesting overnight at room temperature and 1 h at 96 °C, it was diluted 15-20 times with H_2O and filtrated for measurement. UV-vis spectroscopy was recorded on Cary 5000 UV-Vis-NIR instrument from Agilent.

Electrode Preparation:

Normally, to prepare the ink, 10 mg of the catalyst powder was dispersed in 450 µL DMF and sonicated for 20 min, before the addition of 50 µL of 5 wt. % PVDF in DMF solution and further sonication for 20 min. Specifically, for PM-5% Cu NPs+TCNQ900, the ink was prepared by mixing 9.5 mg TCNQ900 and 0.5 mg commercial Cu NPs in 450 µL DMF with 50 µL of 5 wt. % PVDF in DMF solution.

For LSV tests, catalysts were loaded on glassy carbon (GC) electrodes from ALS (6 mm outer diameter, 3 mm inner diameter, surface area of 0.071 cm^2). Before loading procedure, GC electrodes were polished with alumina powder slurries of 0.05 µm. Then, 7 µL of the ink was drop-cast on the GC, resulting in a nominal loading of ~2 mg cm^{-2}.

For bulk electrolysis, the catalyst ink was drop-cast on carbon paper of a 1 cm^2 area (single side). Before deposition, the carbon paper was first washed by sonication successively in

acetone, ethanol, and water, for 20 min each time, and eventually dried at 60 °C overnight. Then, 150 μL of the catalyst ink was drop-cast on one side of the carbon paper under the heating of both irradiation lamp and hotplate, resulting in a loading of ~3 mg cm^{-2}.

Additionally, for Bare Cu NPs electrode, the ink was prepared by dispersing 2 mg of commercial Cu NPs in 1.99 mL DMF with 10 μL of 5 wt. % PVDF in DMF solution. Then, 7 μL of the ink was drop-cast on the GC for LSV tests, resulting in a nominal loading of ~0.1 mg cm^{-2}. For bulk electrolysis, 150 μL of the catalyst ink was drop-cast on one side of the carbon paper under the heating of both irradiation lamp and hotplate, resulting in a loading of ~0.15 mg cm^{-2}.

Electrochemical measurements:

Electrochemical experiments were performed using a Biologic potentiostat (SP-300) and an H-cell. A three-electrode system was assembled using Ag/AgCl in saturated KCl as the reference electrode and graphite as the counter electrode. The working electrode and the reference electrode were put in the cathode chamber, and the counter electrode was in the anode chamber. 20 ml of 1 M NaOH (containing certain amount of $NaNO_2$ and $NaNO_3$) was added into the Cathode compartment, and another 20 mL of 1 M NaOH was added into the Anode compartment. Nafion 117 proton exchange membrane was used as the separator. During the electrochemical tests, the cathode chamber was under constant Ar flow and string of 300 r.p.m. Linear sweep voltammetry (LSV) measurements were carried out at a scan rate of 10 mV s^{-1} without iR compensation, and the corresponding onset potential was defined as the point reaching S/N = 5. Bulk electrolysis was conducted with chronoamperometry method under different potential for 0.5 h, if not mentioned specifically. After bulk electrolysis, the electrolyte in the working chamber was sampled and tested for products detection. All potential values are converted to the reversible hydrogen electrode (RHE), using the following equation:

$$E_{RHE} = E_{Ag/AgCl} + 0.197\ V + 0.059 \times pH - i \times R_u \quad (1)$$

E_{RHE} is the potential *vs.* RHE, $E_{Ag/AgCl}$ is the potential *vs.* the reference electrode Ag/AgCl in saturated KCl, R_u is the uncompensated resistance, i is the current and 0.197 V is the standard potential of the reference electrode.

Isotopic nitrite $^{15}NO_2^-$ labeling experiments:

Such experiments were performed by using $Na^{15}NO_2$ as the isotopic nitrite source. For Cu/TCNQ900-11%, isotopically labeled $^{15}NO_2^-$ at a concentration of 2.5 mM were doped into the 1 M NaOH + 100 mM $Na^{14}NO_3$ electrolyte. Chronoamperometry tests were then performed under – 0.15 V *vs.* RHE. The electrolyte was sampled when certain coulomb (30, 60, 90 C) of electrons transferred on working electrode and the $^{15}NH_3$ *vs.* $^{14}NH_3$ was quantified by ^1H-NMR. For Cu/TCNQ900-1%, the experimental condition was similar except for the concentration of doped $^{15}NO_2^-$ was 0.5 mM.

Determination of ammonia:

Non-isotopically ammonia was quantified by UV–Vis using the Berthelot reaction. Specifically, 0.2 mL of the electrolyte (or diluted electrolyte if needed) was pipetted into a vial containing 1.8 mL of 1 M NaOH. To this, 2 mL of a 1 M NaOH solution that contains 5 wt.% salicylic acid, and 5 wt.% sodium citrate was added. Next, 1 mL of a 0.05 M NaClO and 0.2 mL of a 1 wt.% $C_5FeN_6Na_2O$ (sodium nitroferricyanide) was added to the solution. The solution was incubated in the dark at ambient condition for 1 h, then UV-Vis spectra were recorded. The concentration of NH3 is determined using the maximum absorbance at a 654 nm

wavelength. For isotopic labeling experiments, nuclear magnetic resonance (NMR) spectroscopy was used to detect and quantify both $^{14}NH_3$ and $^{15}NH_3$. Dimethylsulfoxide-d^6 (DMSO) and Maleic acid were used as the locking solvent and internal standard, respectively. For the NMR test solution, 250 μL of the electrolysis electrolyte, 100 μL of DMSO-d^6, 250 μL of 0.55 M H_2SO_4, and 100 μL of 0.5 M MA in DMSO-d^6 were mixed. The NMR spectrum was obtained on an Agilent 400 MHz spectrometer. The signal from H_2O was restrained for better accuracy by applying the solvent suppression method during acquisition. MestReNova software was used to process the NMR data.

Determination of nitrite:

Nitrite was quantified by UV–Vis based on the Griess test. Griess reagent was prepared by mixing p-aminobenzenesulfonamide (4 g), N-(1-Naphthyl) ethylenediamine dihydrochloride (0.2 g), DI-water (50 ml), and phosphoric acid (10 ml, ρ=1.70 g/ml). Then, 100 μL of the electrolyte (or diluted electrolyte if needed) was placed in a vial containing 4.9 mL DI-water. Next, 0.1 ml color reagent was added to the above solution. After setting it for 20 min, the absorption intensity at the wavelength of 540 nm was recorded.

Calculation of the yield and faradaic efficiency:

For the nitrate (NO_3^-) and nitrite (NO_2^-) reduction reactions, the NH_3 Faradaic efficiency (FE) was calculated by Eq. (2).

$$FE_{NH3} = \frac{n * F * c_{NH3} * V}{Q} \quad (2)$$

For the nitrate (NO_3^-) and nitrite (NO_2^-) reduction reactions, the partial current density of NH_3 (j_{NH3}) was calculated by Eq. (3).

$$j_{NH3} = \frac{FE_{NH3} * Q}{t} \quad (3)$$

In the NO_3^-RR, the nitrate (NO_3^-) to nitrite (NO_2^-) reduction Faradaic efficiency was calculated by Eq. (4).

$$FE_{NO2-} = \frac{n * F * c_{NO2-} * V}{Q} \quad (4)$$

where n is the number of electrons transferred (for NO_3^- to NO_2^- is n = 2, for NO_3^- to NH_3 is n = 8, and for NO_2^- to NH_3 is n = 6), F is Faraday's constant (96,485 C mol^{-1}), c_{NH3} is the mole concentration of NH_3(aq), V is the volume of the electrolyte, Q is the total charge transferred in electrolysis, t is the duration of the chronoamperometric measurement, c_{NO2-} is the mole concentration of NO_2^-.

Supplementary Note 1.

Characterization of TCNQ900

After thorough washing with 1 M HCl and deionized water, the resulting material exhibited characteristic XRD features (**Fig. S1**) of highly porous carbon, including two broad reflections at $2\theta \approx 24^{\circ}$ and 44°, corresponding to turbostratic carbon ordering, and a sloping baseline at low angles indicative of a highly developed surface area. Nitrogen sorption analysis confirmed a high BET surface area of 1690 m^2 g^{-1}, with the isotherm revealing the presence of both micropores and small mesopores (**Fig. S2**). Elemental analysis showed a composition of 87 wt.% C, 7 wt.% N, and 1 wt.% H (**Fig. S3**), with the remaining mass attributed to adsorbed moisture, according to the previous study.[1] ICP-OES revealed the presence of 0.76 wt.% of residual Zn species (**Fig. S3**), presumably strongly coordinated through nitrogen atoms. As shown in **Fig. S4a**, X-ray photoelectron spectroscopy (XPS) of C 1*s* confirmed the successful integrating of N into graphitic carbon matrix, showing typical features of C bonded with N between 286 eV ~ 290 eV. XPS of N 1*s* (**Fig. S4b**) further revealed a complex nitrogen speciation pattern, with at least four distinct components typically assigned to pyridinic (398.4 eV), pyrrolic (400.7 eV), graphitic (402.0 eV), oxidized nitrogen (403.4 eV).[1] High-resolution TEM and EDX elemental mapping confirmed the homogeneous distribution of carbon and nitrogen throughout the material (**Fig. S5**). In summary, and consistent with our prior work, thermal treatment at 900 °C enables the retention of a substantial nitrogen content while achieving high electronic conjugation, offering a favorable balance for electrochemical applications.

Supplementary Figures

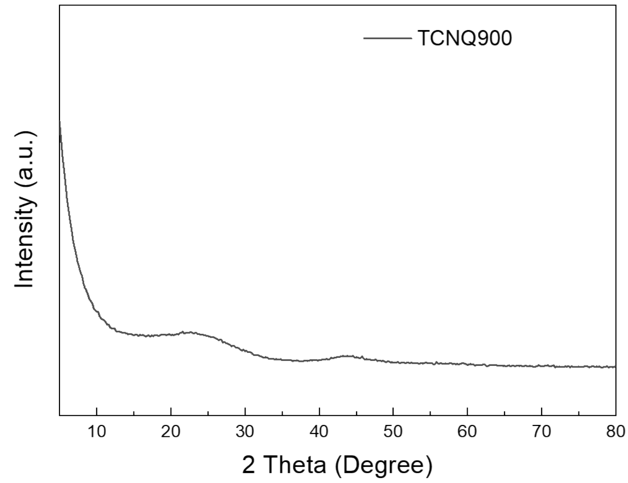

Fig. S1 X-ray diffraction patterns of as-synthesized TCNQ900 samples.

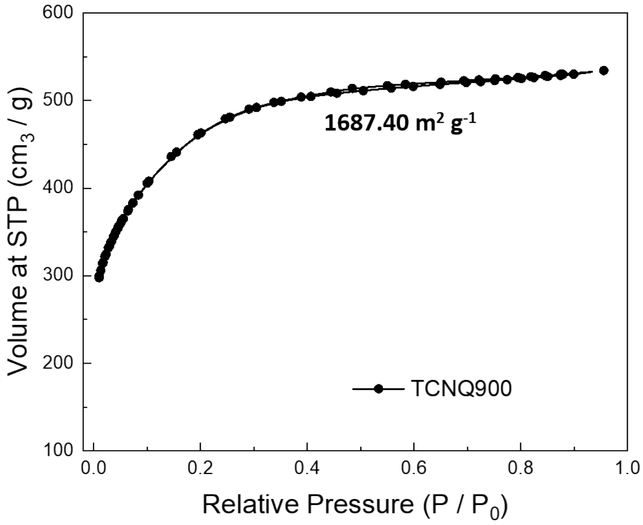

Fig. S2 N_2 sorption isotherm at 77 K of TCNQ900. Multipoint Brunauer-Emmett-Teller (BET) analysis on N_2 sorption isotherm showed a specific surface area (SSA) of 1687.4 m^2 g^{-1} of TCNQ900.

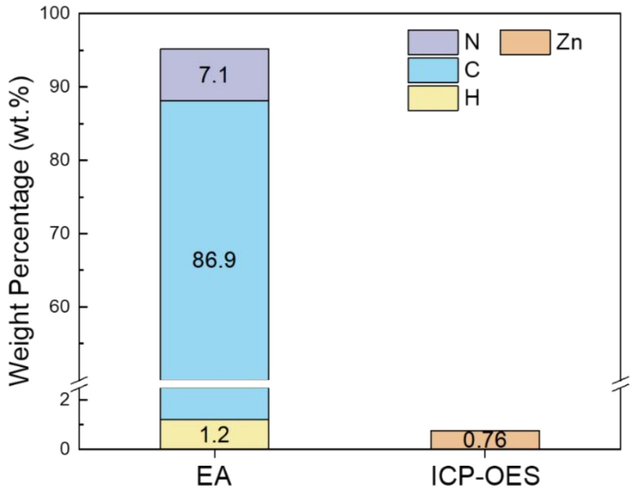

Fig. S3 Elemental Composition of TCNQ900 from elemental analysis and ICP-OES.

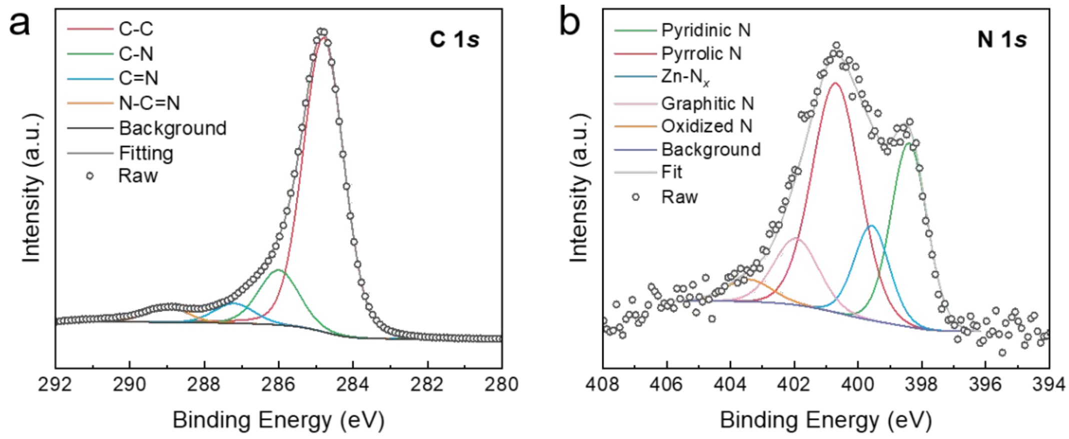

Fig. S4 High resolution XPS of TCNQ900. **a** C 1*s* spectrum, and **b** N 1*s* spectrum.

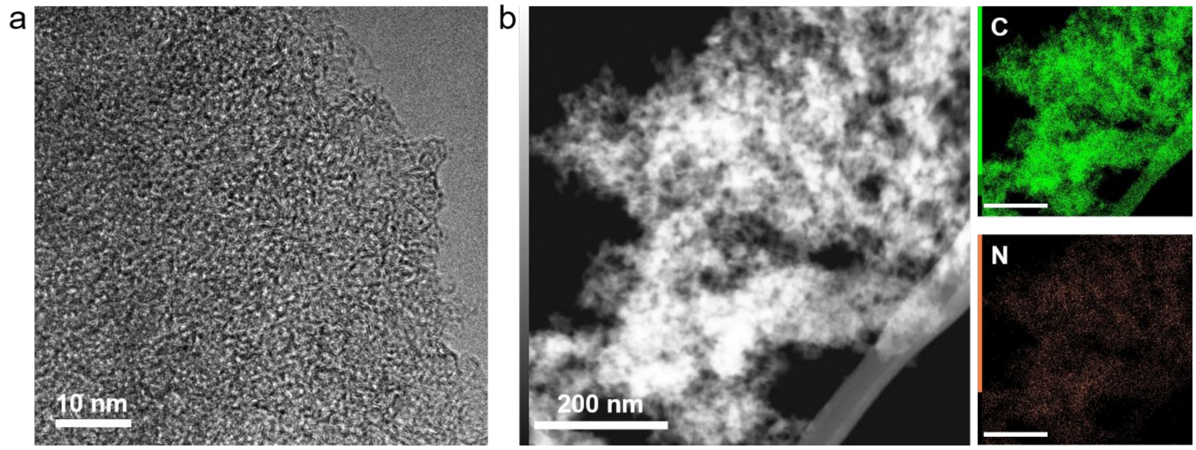

Fig. S5 TEM & EDS-mapping of TCNQ900. **a** High resolution-TEM image of TCNQ900, **b** Energy dispersive X-ray spectrometry (EDX) elemental mapping of TCNQ900.

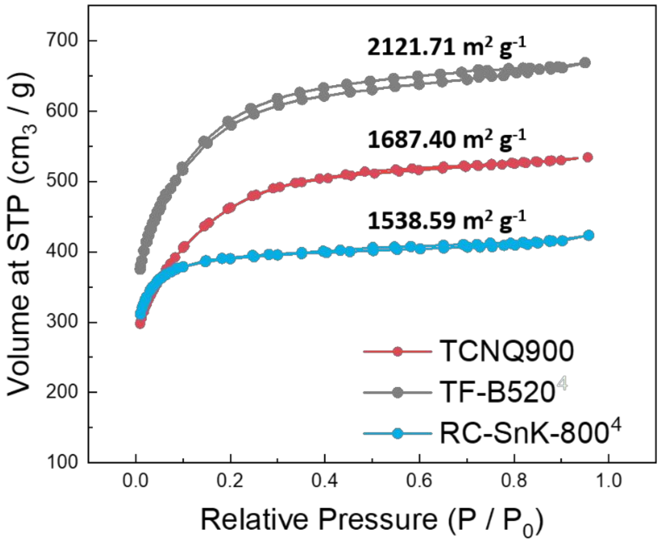

Fig. S6 N_2 sorption isotherm at 77 K of TCNQ900, TF-B520, and RC-SnK-800[4]. BET analysis on N_2 sorption isotherm showed very high specific surface area (SSA) for all three samples. Even though possessing a higher SSA, TF-B520 still presented very limited performance in LSV tests, again indicating the key role of N-dopants in NO_2^-RR.

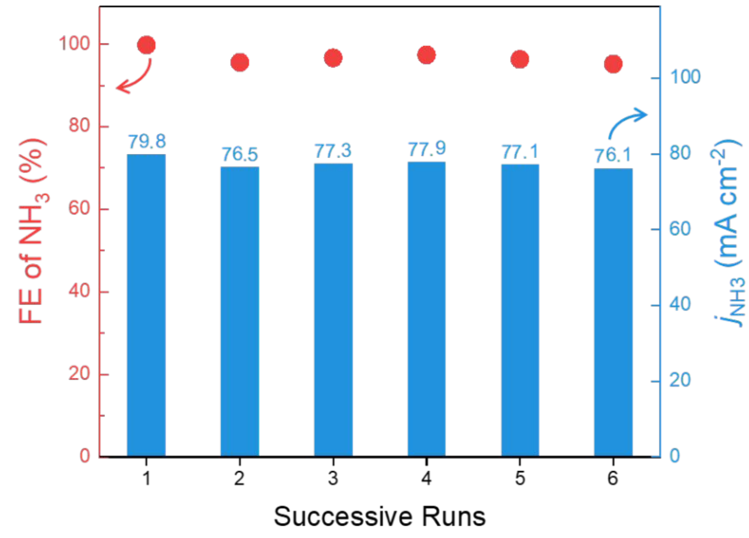

Fig. S7 Successive runs of TCNQ900 in bulk electrolysis showing great stability for NO_2^-RR.

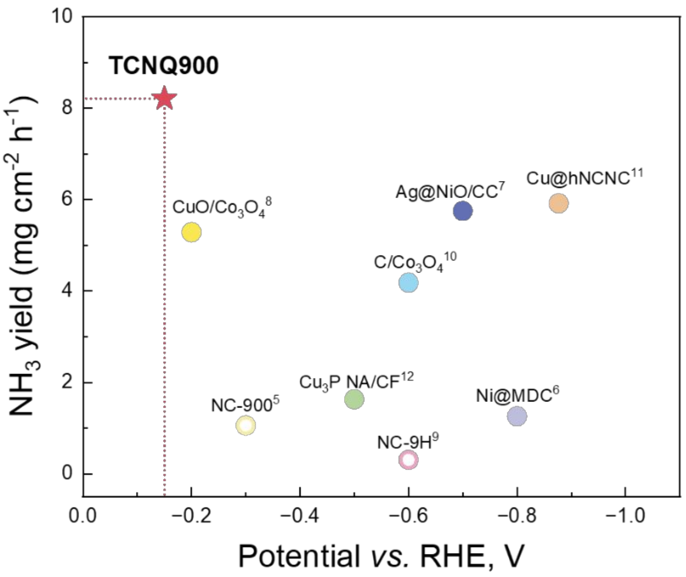

Fig. S8 Comparison of NO_2^-RR performance between TCNQ900 and published works[5-12].

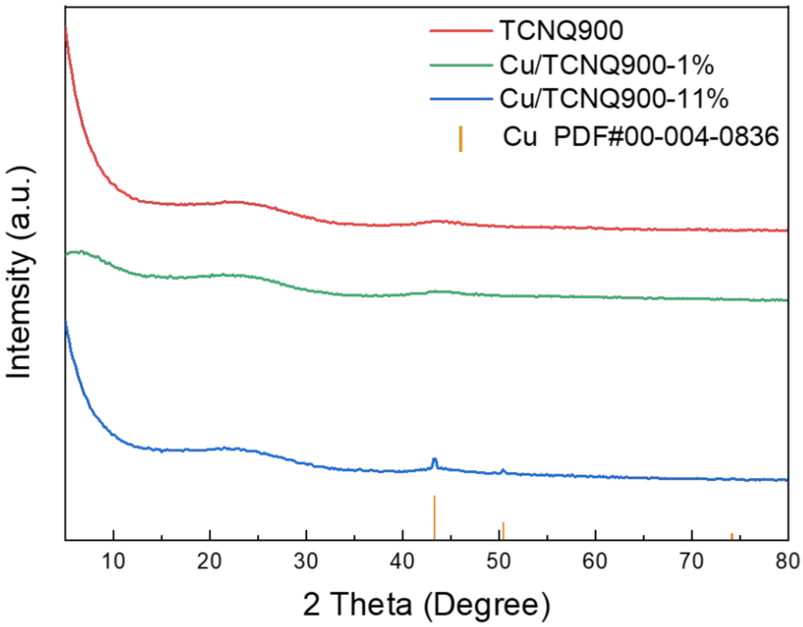

Fig. S9 XRD patterns of TCNQ900, Cu/TCNQ900-1%, and Cu/TCNQ900-11%.

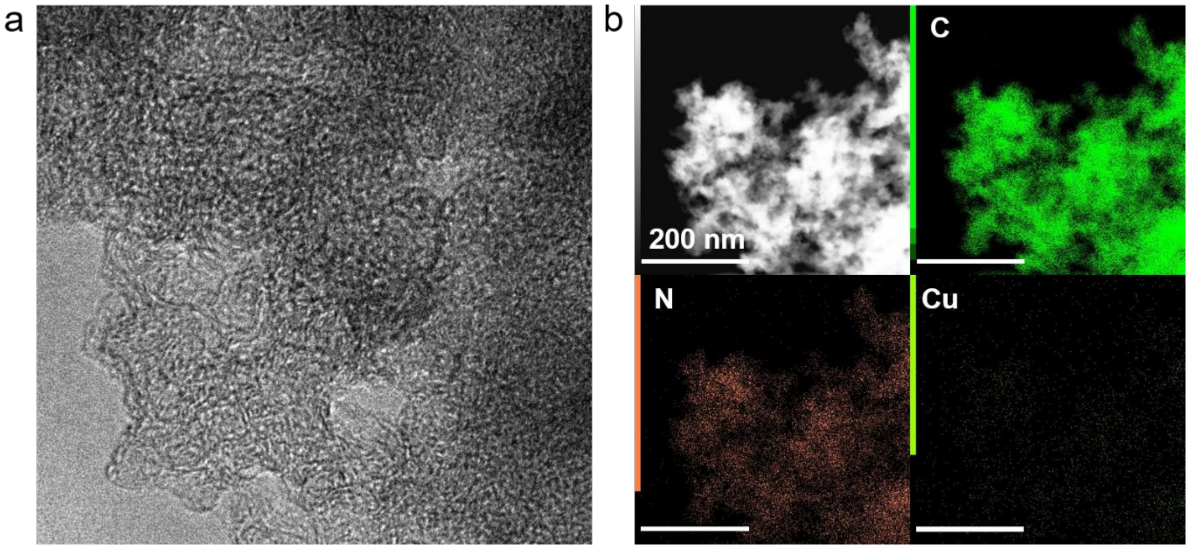

Fig. S10 TEM & EDS-mapping of Cu/TCNQ900-1%. **a** High resolution-TEM image, **b** EDX elemental mapping of C, N, and Cu.

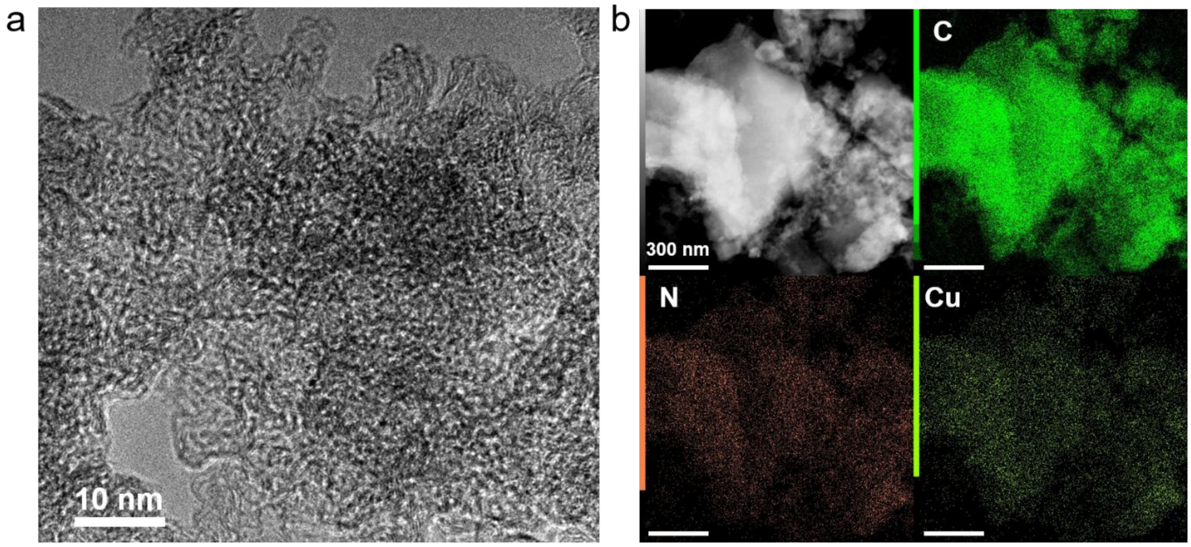

Fig. S11 TEM & EDS-mapping of Cu/TCNQ900-11%. **a** High resolution-TEM image, **b** EDX elemental mapping of C, N, and Cu.

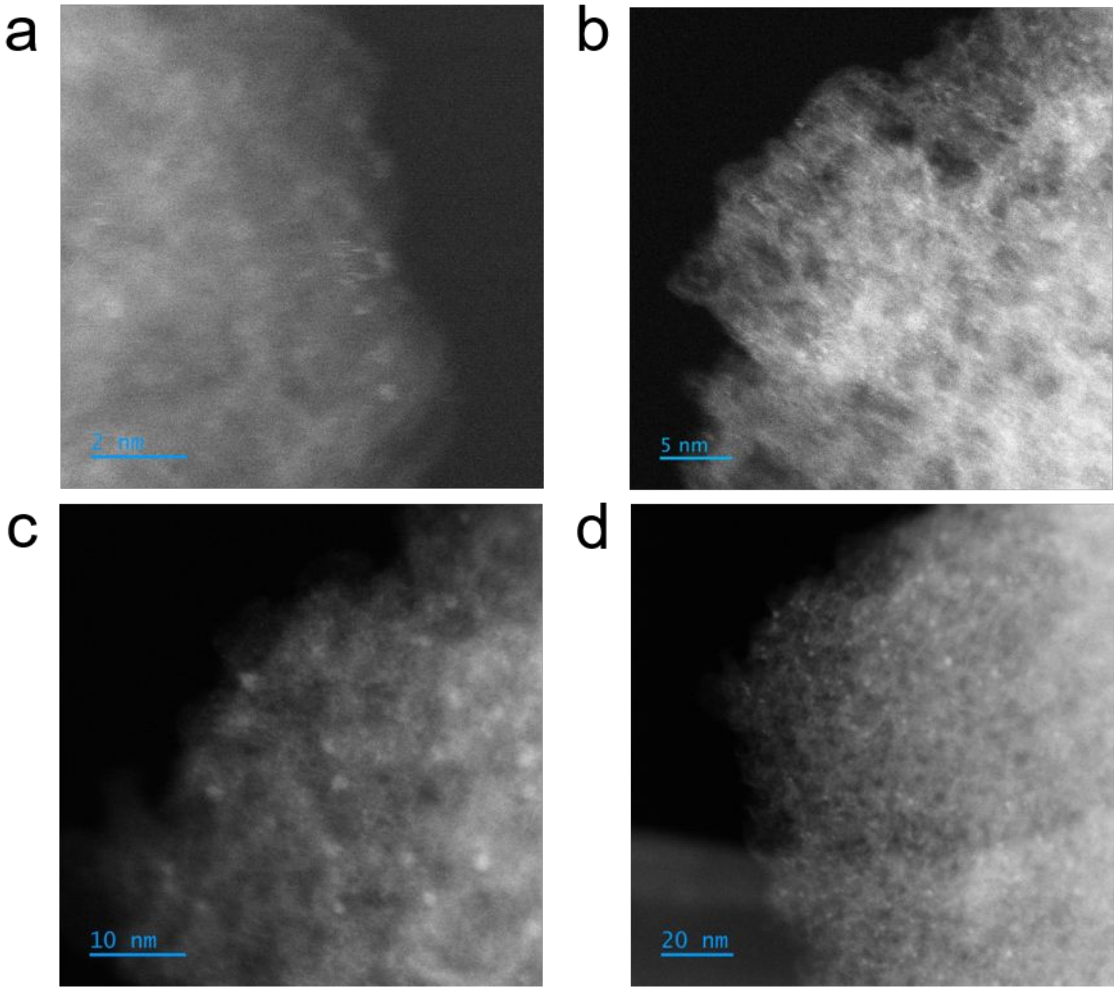

Fig. S12 HAADF-STEM images of Cu/TCNQ900-1% (**a**,**b**) and Cu/TCNQ900-11% (**c**,**d**).

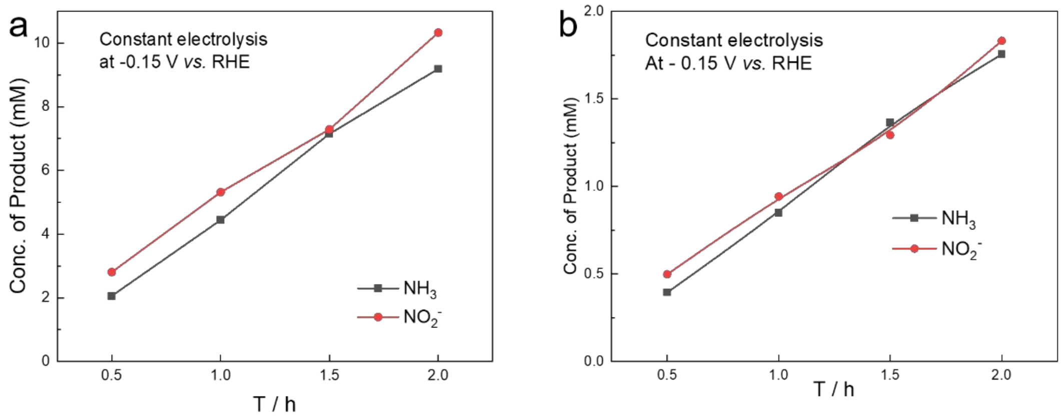

Fig. S13 Concentration of product – Time curve during constant electrolysis with Cu/TCNQ900-11% (a) and Cu/TCNQ900-1% (b) at – 0.15 V *vs.* RHE in 1 M NaOH + 100 mM $NaNO_3$.

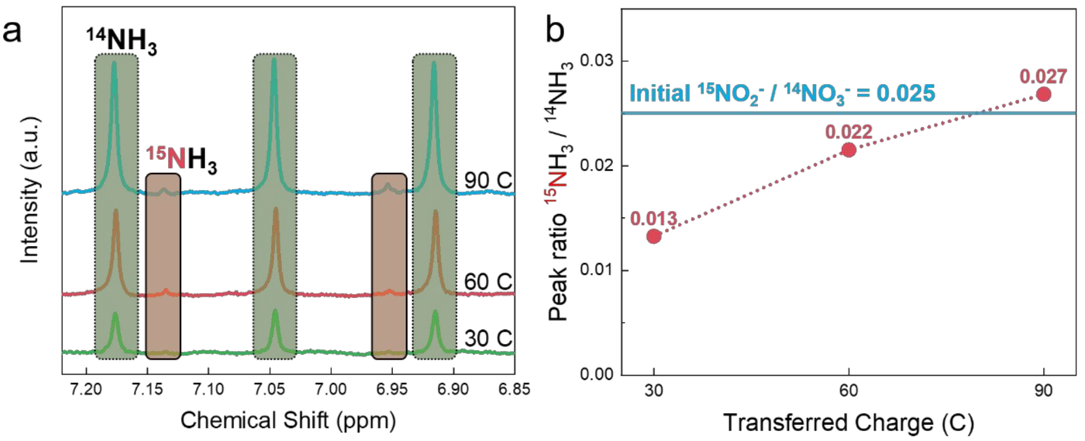

Fig. S14 a ^{1}H-NMR spectra after constant electrolysis with Cu/TCNQ900-11% at – 0.15 V *vs.* RHE in doping experiment. **b** Corresponding peak ratio of $^{15}NH_3/^{14}NH_3$ at each sampled point.

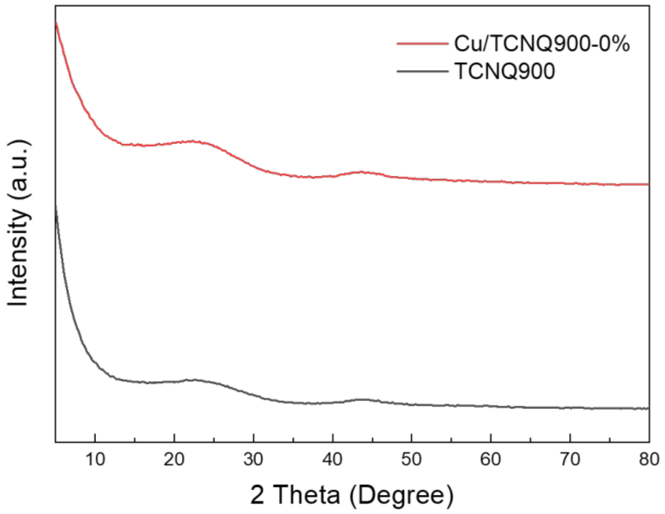

Fig. S15 Comparison of XRD patterns between TCNQ900 and Cu/TCNQ900-0%.

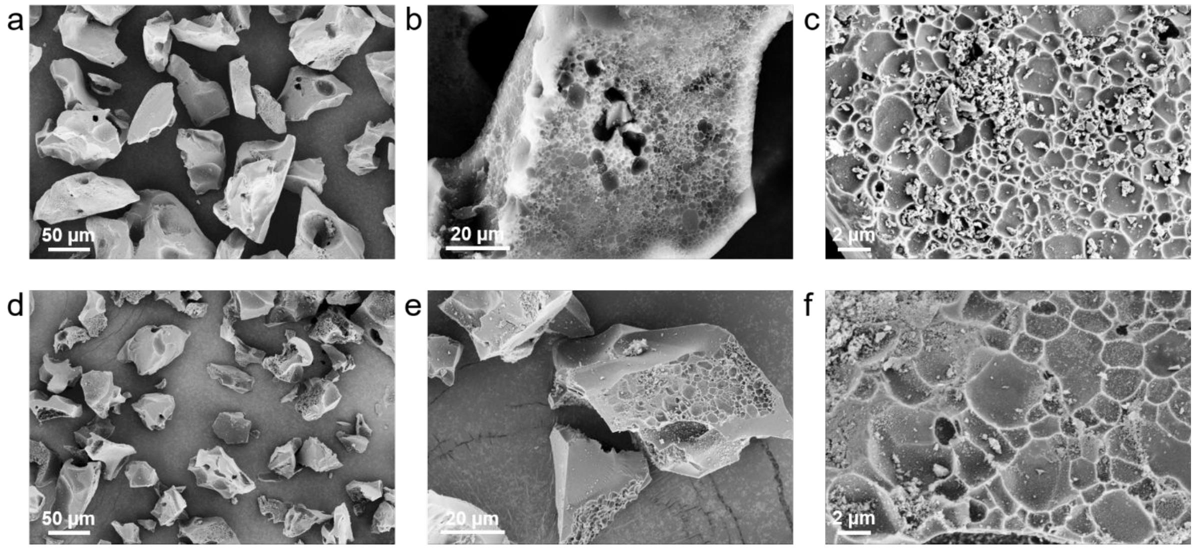

Fig. S16 Comparison of SEM images under various resolutions between (**a-c**) Cu/TCNQ900-0% and (**d-f**) TCNQ900.

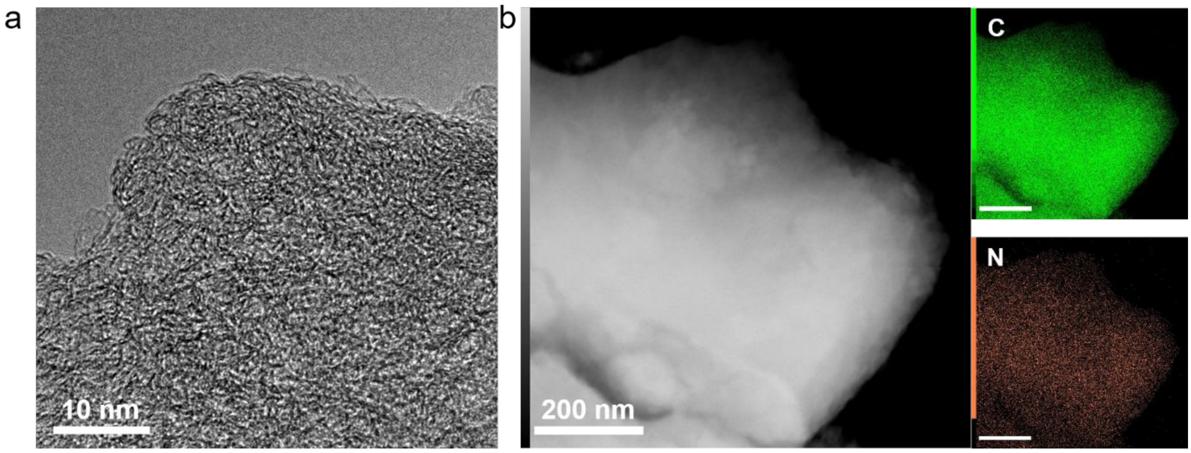

Fig. S17 TEM & EDS-mapping of Cu/TCNQ900-0%. **a** High resolution-TEM image, **b** EDX elemental mapping of C, N, and Cu.

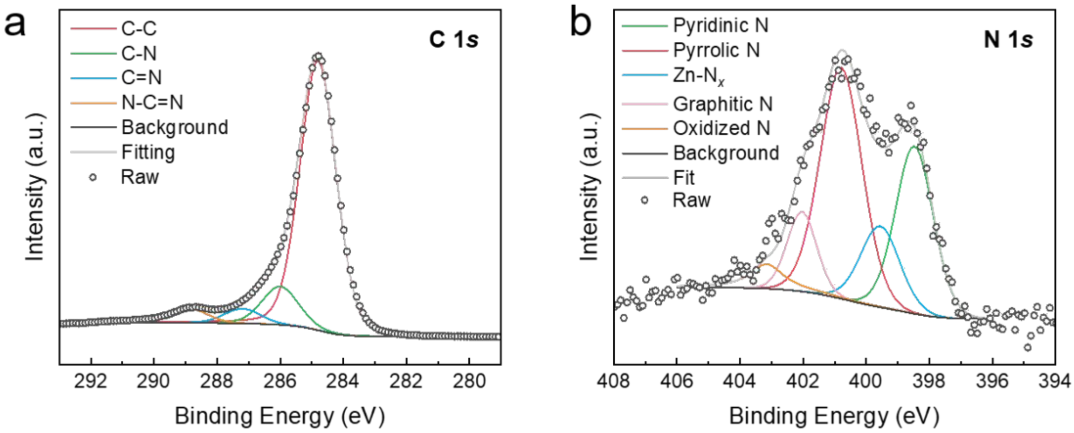

Fig. S18 High resolution XPS of Cu/TCNQ900-0%. **a** C 1*s* spectrum, and **b** N 1*s* spectrum.

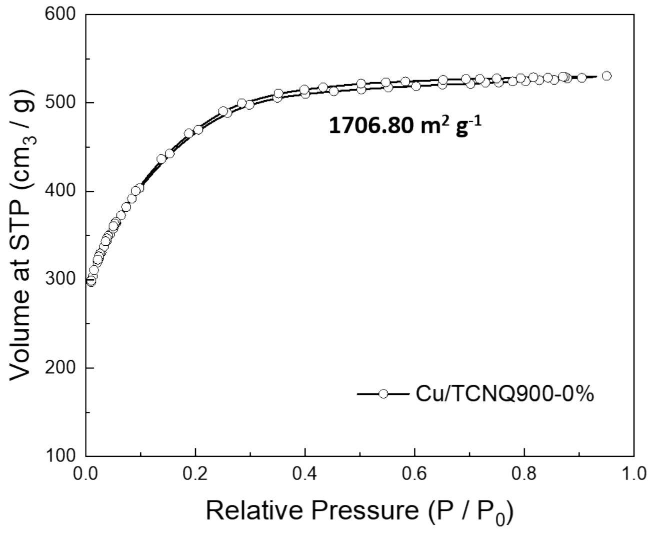

Fig. S19 N_2 sorption isotherm at 77 K of Cu/TCNQ900-0%. Such isotherm of Cu/TCNQ900-0% presented very similar properties to TCNQ900, and multipoint BET analysis further confirmed a similar SSA of TCNQ900, indicating that the pyrolysis and washing steps involved in the synthesis of Cu/TCNQ900 samples wouldn't greatly change the porous structure of TCNQ900.

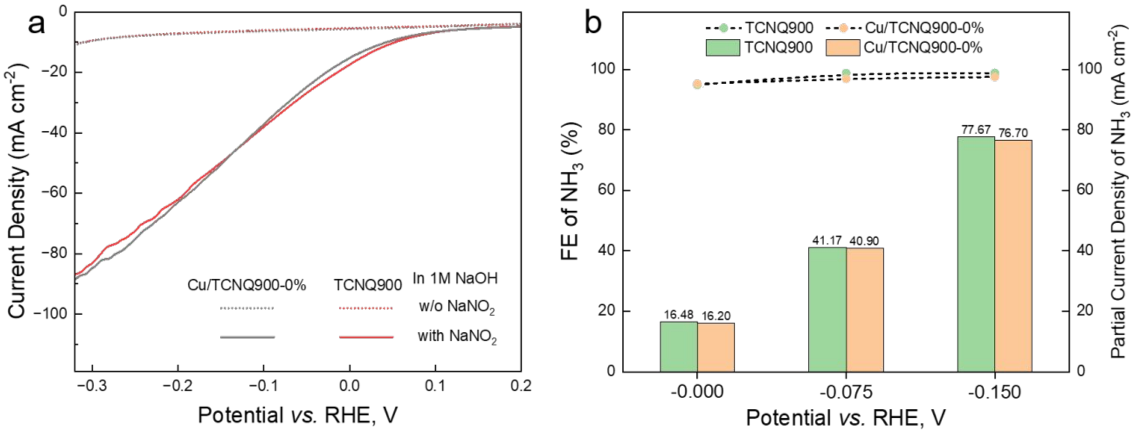

Fig. S20 Comparison of NO_2^-RR performance between TCNQ900 and Cu/TCNQ900-0%. **a** LSV curves in 1 M NaOH with and without 100 mM $NaNO_2$, **b** Bulk electrolysis performance in 1 M NaOH + 100 mM $NaNO_2$ under different potentials.

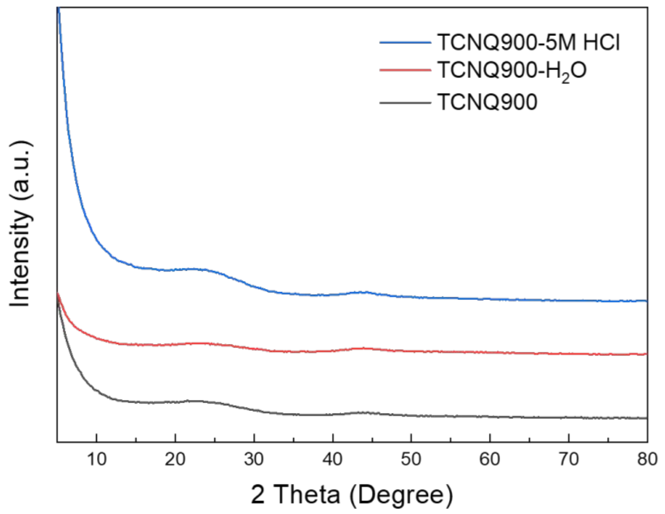

Fig. S21 XRD patterns of TCNQ900, TCNQ900-H_2O, and TCNQ900-5M HCl. Though Zn residue contents varied in three samples due to different washing conditions, no obvious intensity peaks corresponding to Zn-related species could be seen for all three samples, which could be due to the intendency to form highly dispersed Zn-N_x structure during high temperature pyrolysis.

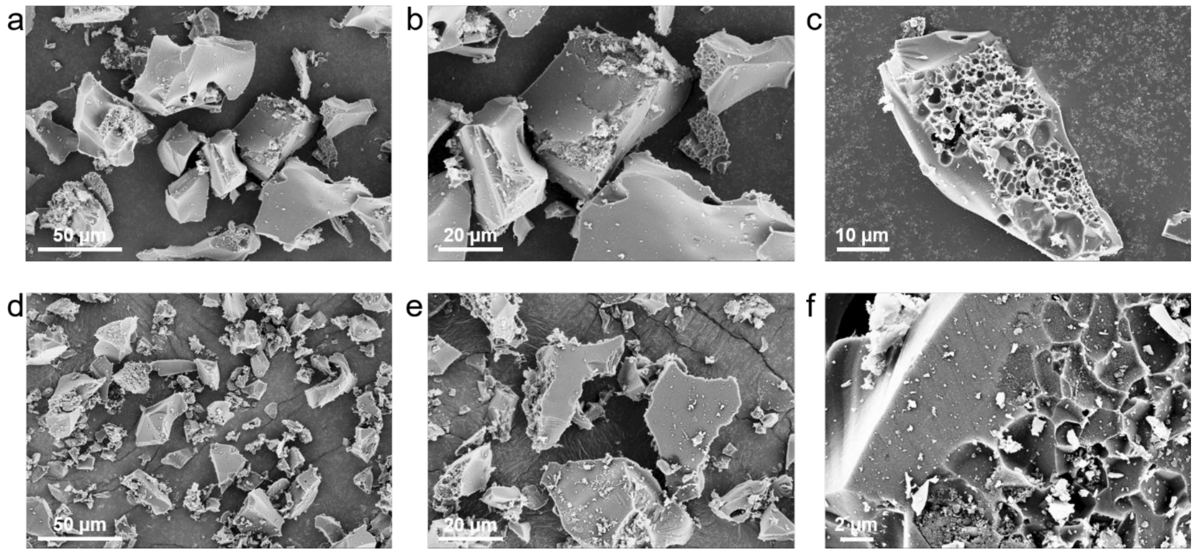

Fig. S22 Comparison of SEM images under various resolutions between (**a-c**) TCNQ900-H_2O and (**d-f**) TCNQ900-5M HCl. Both samples showed very similar morphology to TCNQ900, indicating the acid concentration and Zn residue wouldn't affect the overall morphology.

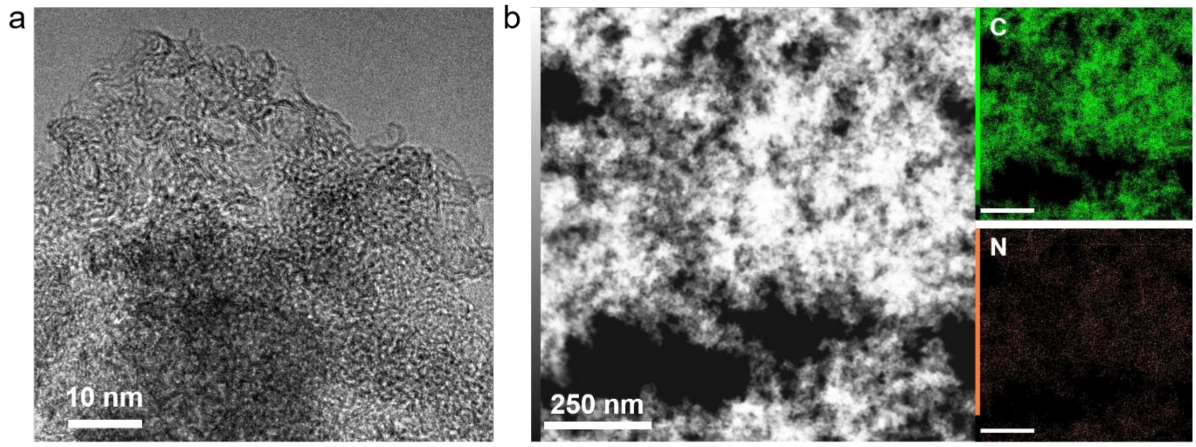

Fig. S23 TEM & EDS-mapping of TCNQ900-H_2O. **a** High resolution-TEM image of TCNQ900-H_2O, **b** Energy dispersive X-ray spectrometry (EDX) elemental mapping of TCNQ900-H_2O.

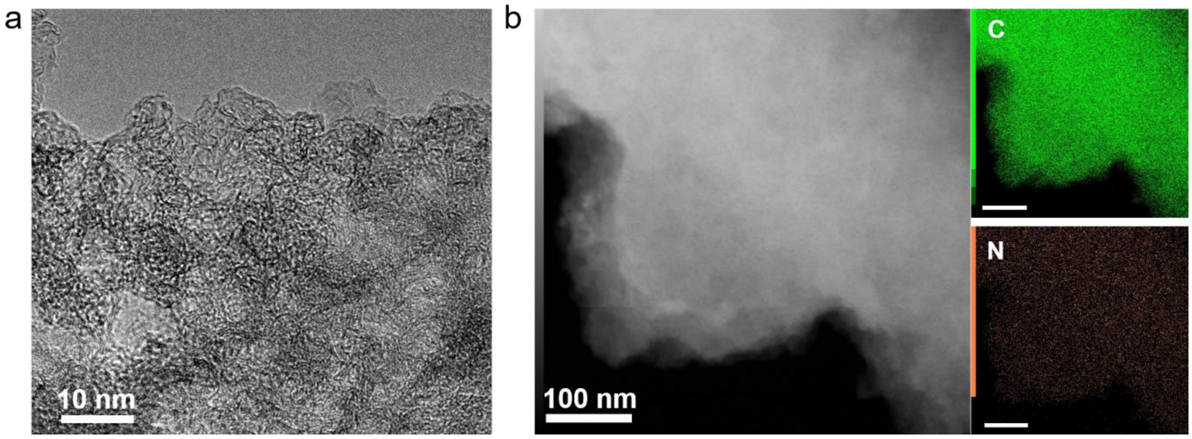

Fig. S24 TEM & EDS-mapping of TCNQ900-5M HCl. **a** High resolution-TEM image of TCNQ900-5M HCl, **b** Energy dispersive X-ray spectrometry (EDX) elemental mapping of TCNQ900-5M HCl.

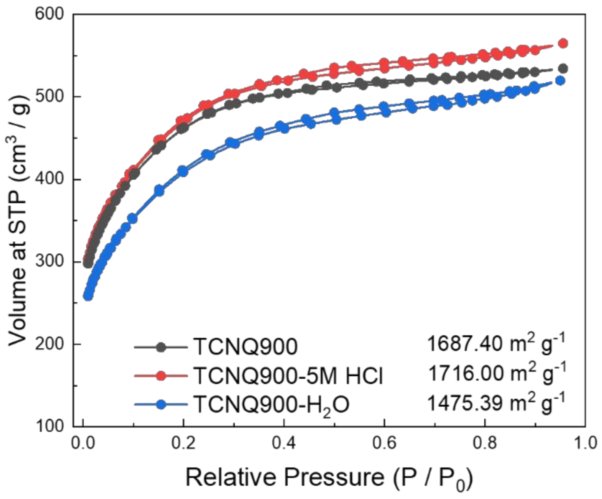

Fig. S25 N_2 sorption isotherms at 77K of TCNQ900, TCNQ900-H_2O, and TCNQ900-5M HCl.

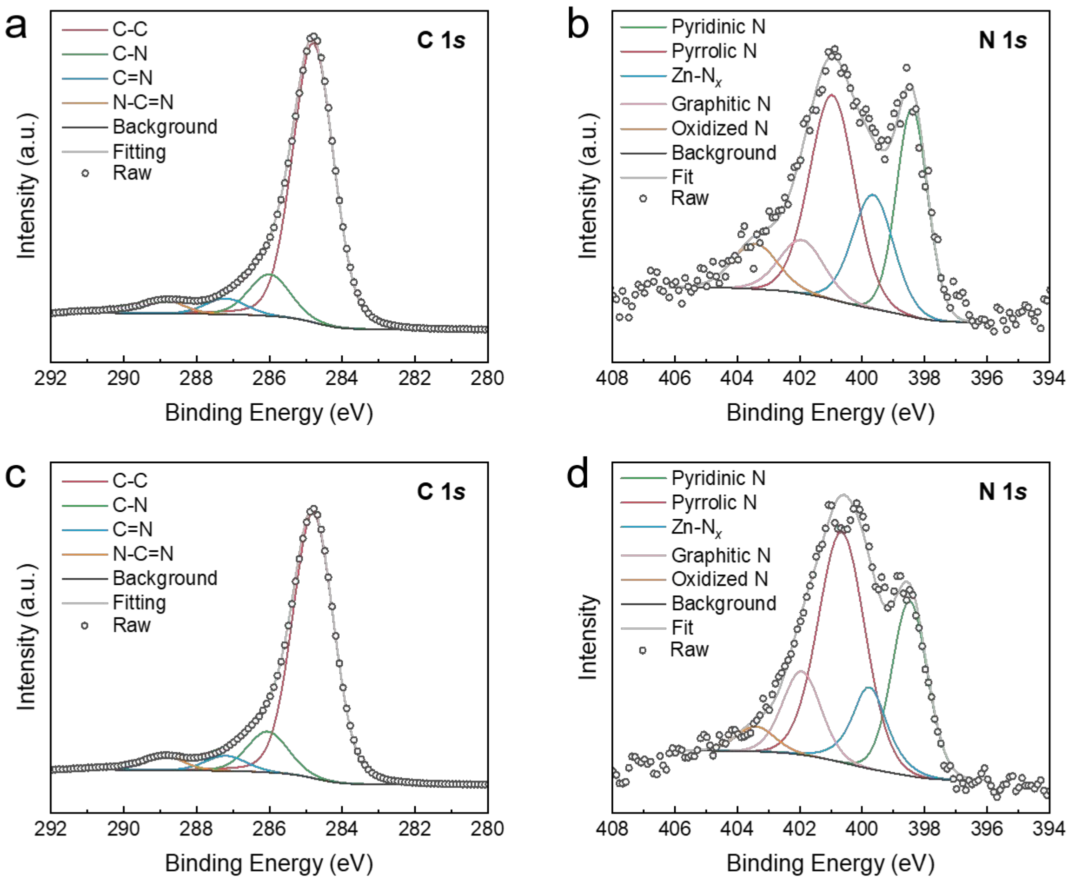

Fig. S26 High resolution XPS of TCNQ900 under different wash condition. **a** C 1*s* spectrum, and **b** N 1*s* spectrum of TCNQ900-H_2O. **c** C 1*s* spectrum, and **d** N 1*s* spectrum of TCNQ900-5M HCl.

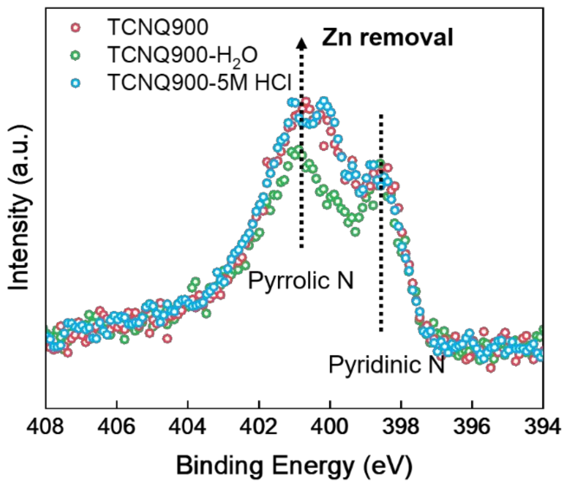

Fig. S27 Comparison of N 1s spectra of TCNQ900, TCNQ900-H_2O, and TCNQ900-5M HCl.

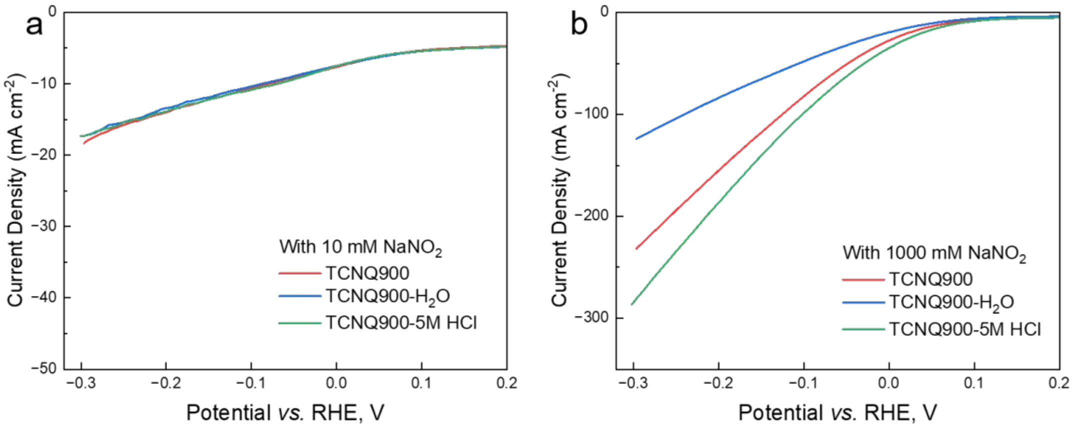

Fig. S28 Comparison of LSV curves between TCNQ900, TCNQ900-H_2O, and TCNQ900-5M HCl in 1 M NaOH with different concentrations of $NaNO_2$. **a** in 1 M NaOH + 10 mM $NaNO_2$. **b** in 1 M NaOH + 1000 mM $NaNO_2$.

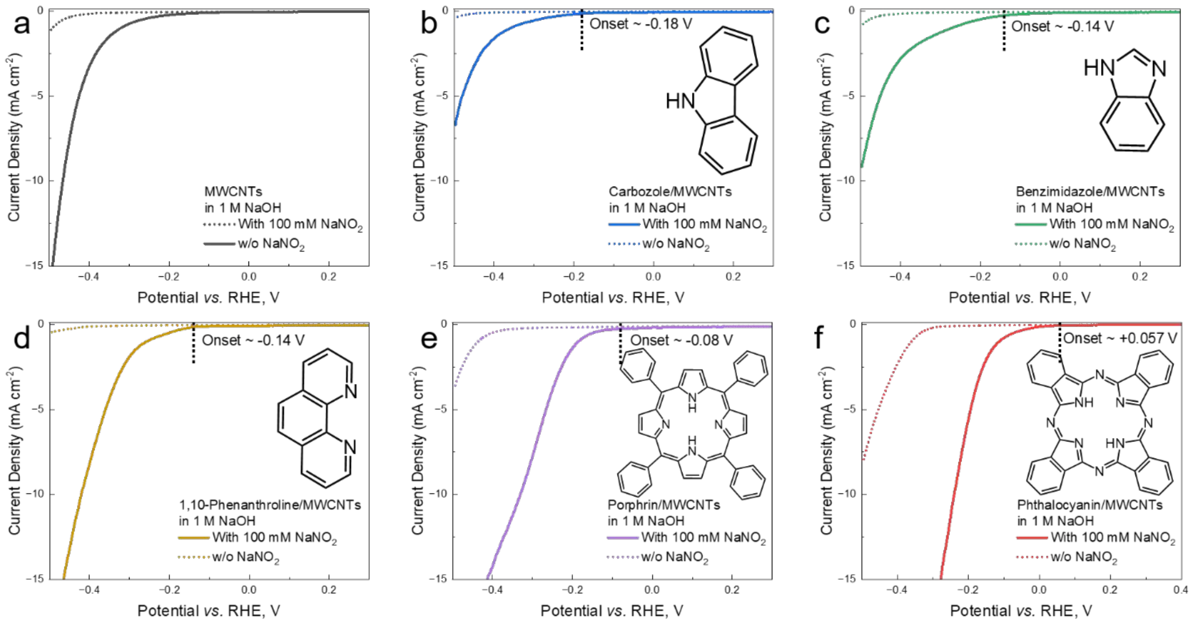

Fig. S29 LSV curves in 1 M NaOH with and without 100 mM $NaNO_2$ on different model catalysts. **a** pristine-MWCNTs, **b** Carbozole/MWCNTs, **c** Benzimidazole/ MWCNTs, **d** 1,10-Phenanthroline/MWNCTs, **e** Porphrin/MWNCTs, **f** Phthalocyanin/MWCNTs. The onset potential for each model catalyst was defined as the point in LSV reaching S/N = 5.

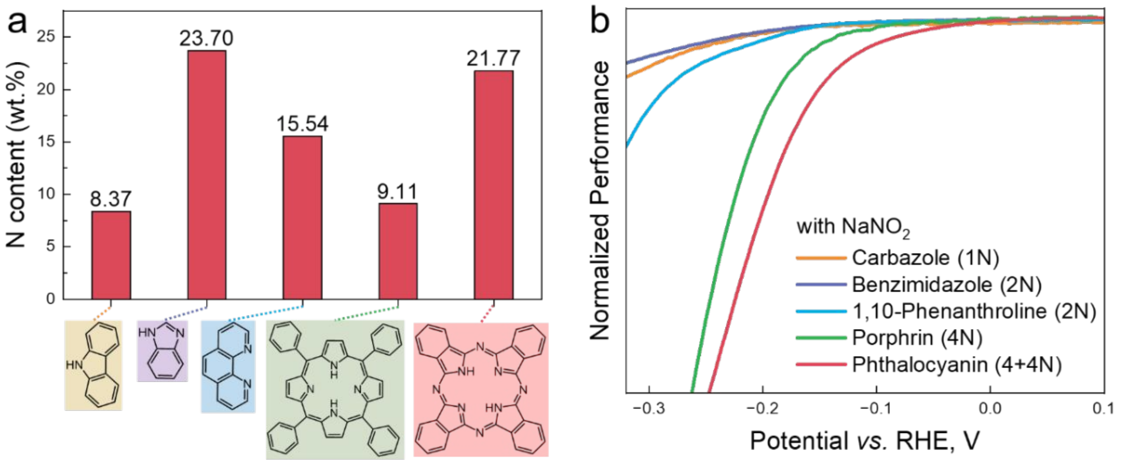

Fig. S30 a N content of different model molecules, **b** Normalized LSV curves in **Fig. 4b** with N content of different model molecules in 1 M NaOH with and without 100 mM $NaNO_2$. To account for slight differences in overall nitrogen loading, we suspect a minimal weight loss during the loading and each loaded molecule contribute equally, and then normalized those LSV curves with N-content within each model molecules. As shown in **Fig. S30 b**, normalized performance still confirmed the superiority of model molecules containing more confined N cavity.

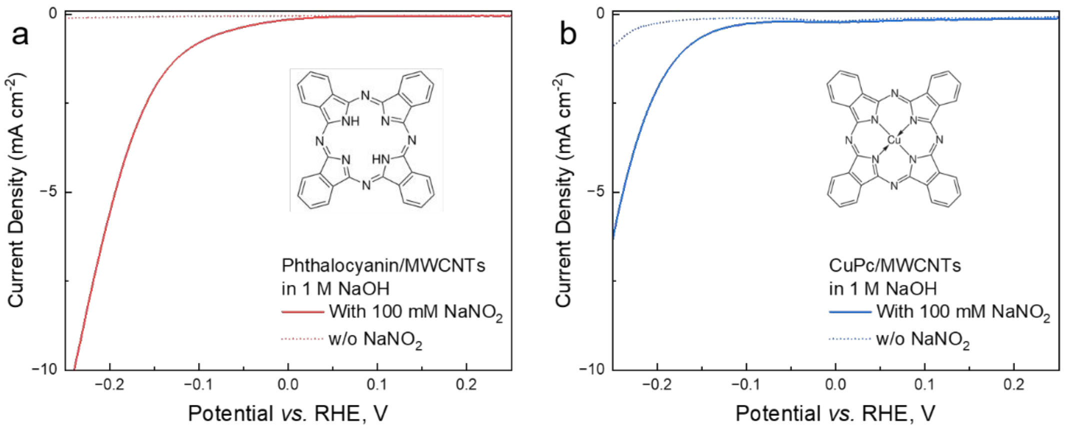

Fig. S31 Comparison of LSV curves in 1 M NaOH with and without 100 mM $NaNO_2$ on Phthalocyanin/MWCNTs (a) and CuPc/MWCNTs (b).

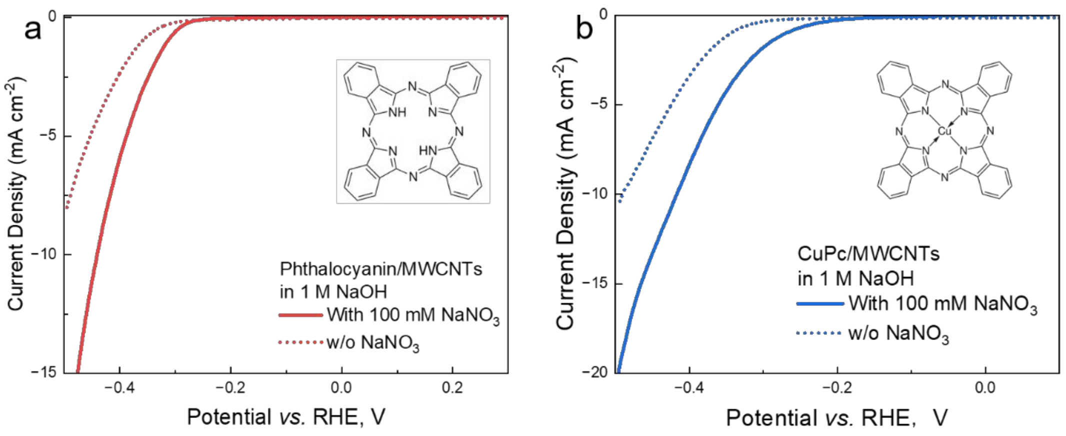

Fig. S32 Comparison of LSV curves in 1 M NaOH with and without 100 mM $NaNO_3$ on Phthalocyanin/MWCNTs (a) and CuPc/MWCNTs (b).

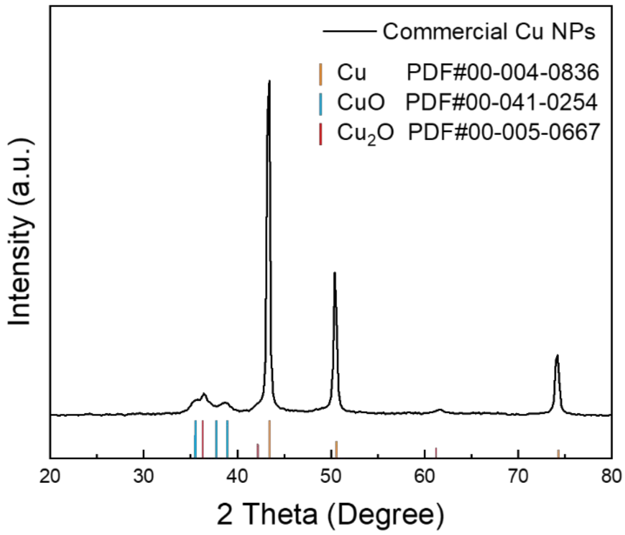

Fig. S33 XRD pattern of Commercial Cu NPs. Such Cu NPs are prone to be partially oxidized in air due to its small particle size, thusly small peaks assigned to CuO_x can be observed from XRD. That is also consistent with the supplier's specification indicating the presence of <10% oxygen.

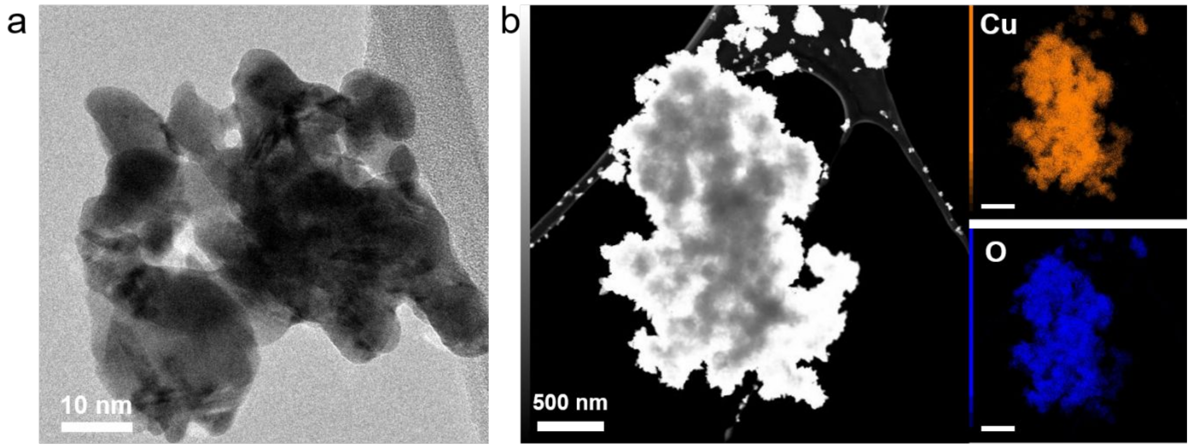

Fig. S34 TEM & EDS-mapping of Commercial Cu NPs. **a** High resolution-TEM image, and **b** EDX elemental mapping of Commercial Cu NPs. TEM image showed the small particle size of such commercial Cu NPs, and EDX mapping further confirmed that Cu NPs are partially oxidized, showing good alignment with XRD result.

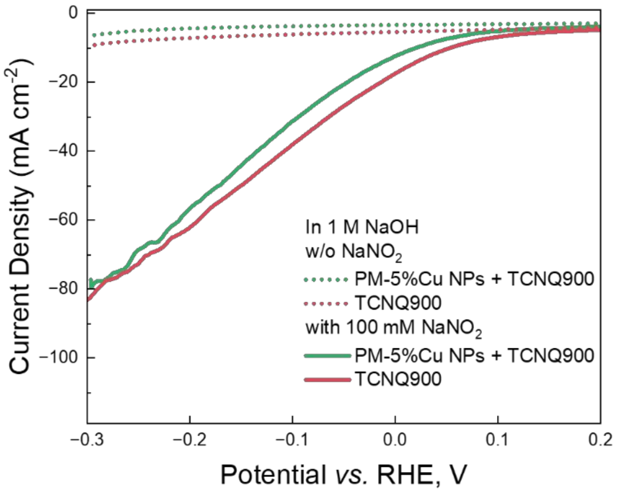

Fig. S35 Comparison of LSV curves in 1 M NaOH with and without 100 mM $NaNO_2$ on pristine TCNQ900 and PM-5% Cu NPs + TCNQ900.

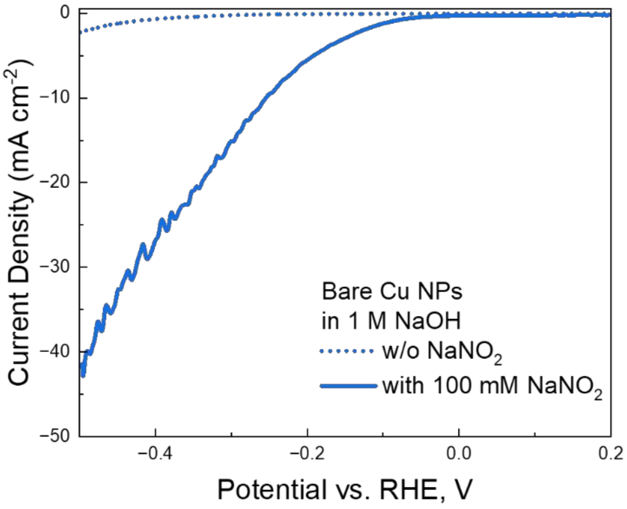

Fig. S36 LSV curves in 1 M NaOH with and without 100 mM $NaNO_2$ on bare Cu NPs.

Table S1. Elemental Analysis results of H, C, N, and O for different samples

Sample	H, wt.%	C, wt. %	N, wt.%	O, wt.%
TCNQ900	1.2	86.9	7.1	/
TCNQ900-H_2O	2.7	75.2	5.2	/
TCNQ900-5M HCl	2.3	73.1	5.5	/
RC-SnK-800[4]	1.0	93.0	/	6.0
Cu/TCNQ900-0%	1.1	88.6	7.2	/
Cu/TCNQ900-1%	3.0	73.4	5.5	/
Cu/TCNQ900-11%	2.9	65.6	5.6	/

Table S2. Variation of main N-species from XPS N 1*s* within different samples

Sample	Pyridinic	Pyrrolic	Zn-N_x	Graphitic	Oxidized
	at. %	at. %	at. %	at. %	at. %
TCNQ900	26.4	44.0	13.7	11.9	4.0
TCNQ900-H_2O	26.1	37.2	18.8	9.6	8.3
TCNQ900-5M HCl	25.3	44.5	12.9	13.3	4.0

Table S3. Comparison of NO_2^-RR performance between TCNQ900 and published works.

Catalyst	Electrolyte	Potential V, vs. RHE	j_{NH3} mA cm^{-2}	FE_{NH3} %	Ref

**TCNQ900**	**1 M NaOH + 100 mM $NaNO_2$**	**-0.15**	**77.70**	**98.80**	**This work**
NC-900	0.5 M K_2CO_3 + 300 mM KNO_2	-0.30	10	50	**5**
Ni@MDC	0.1 M NaOH + 100 mM $NaNO_2$	-0.80	11.93	65.4	**6**
Ag@NiO/CC	0.1 M NaOH + 100 mM $NaNO_2$	-0.7	54.46	90	**7**
CuO/Co_3O_4	1 M KOH + 100 mM $NaNO_2$	-0.2	50	95	**8**
NC-9H	0.05 M PBS + 10 mM KNO_2	-0.60	2.90	72	**9**
C/Co_3O_4	0.5 M K_2SO4 + 50 mM KNO_2	-0.60	39.5	100	**10**
Cu@hNCNC	0.5 M Na_2SO4 + 50 mM $NaNO_2$	-0.88	55.96	94	**11**
Cu_3P NA/CF	0. 1 M PBS + 100 mM $NaNO_2$	-0.50	15.38	91.2	**12**

Table S4. ICP results of Cu and Zn contents for different samples

**Sample**	**Cu, wt.%**	**Zn, wt.%**
Cu/TCNQ900-1%	0.79	/
Cu/TCNQ900-11%	5.68	/
CuPc/MWCNTs	1.38	/
TCNQ900	/	0.76
TCNQ900-H_2O	/	2.17
TCNQ900-5M HCl	/	0.44

Reference

1 Grammenos, A. O. *et al.* Harnessing the Electrochemical Hydrogen Storage Capability of N-Doped Carbons for Metal-Free Hydrogenations. *ACS catalysis* **15**, 4519-4532 (2025).

2 Hai, X. *et al.* Scalable two-step annealing method for preparing ultra-high-density single-atom catalyst libraries. *Nature nanotechnology* **17**, 174-181 (2022).

3 Wu, Y., Jiang, Z., Lu, X., Liang, Y. & Wang, H. Domino electroreduction of CO2 to methanol on a molecular catalyst. *Nature* **575**, 639-642 (2019).

4 Zheng, X. *et al.* Tin (II) Chloride Salt Melts as Non - Innocent Solvents for the Synthesis of Low - Temperature Nanoporous Oxo - Carbons for Nitrate Electrochemical Hydrogenation. *Advanced Materials* **36**, 2311575 (2024).

5 Lu, X. *et al.* Multiple Secondary Bond-Mediated C–N Coupling over N-Doped Carbon Electrocatalysts. *Journal of the American Chemical Society* **147**, 19342-19352 (2025).

6 He, X. *et al.* Ambient electroreduction of nitrite to ammonia over Ni nanoparticle supported on molasses-derived carbon sheets. *ACS Applied Nano Materials* **5**, 14246-14250 (2022).

7 Liu, Q. *et al.* Nitrite reduction over Ag nanoarray electrocatalyst for ammonia synthesis. *Journal of Colloid and Interface Science* **623**, 513-519 (2022).

8 Niu, Z., Fan, S., Li, X. & Chen, G. Electrocatalytic Co - Upcycling of Nitrite and Ethylene Glycol over Cobalt–Copper Oxides. *Advanced Energy Materials* **14**, 2303515 (2024).

9 Murphy, E. *et al.* Elucidating electrochemical nitrate and nitrite reduction over atomically-dispersed transition metal sites. *Nature communications* **14**, 4554 (2023).

10 Zhang, R. *et al.* A Zn–nitrite battery as an energy-output electrocatalytic system for high-efficiency ammonia synthesis using carbon-doped cobalt oxide nanotubes. *Energy & Environmental Science* **15**, 3024-3032 (2022).

11 Shen, Z. *et al.* Self-enhanced localized alkalinity at the encapsulated Cu catalyst for superb electrocatalytic nitrate/nitrite reduction to NH3 in neutral electrolyte. *Science Advances* **10**, eadm9325 (2024).

12 Liang, J. *et al.* High-efficiency electrochemical nitrite reduction to ammonium using a Cu 3 P nanowire array under ambient conditions. *Green Chemistry* **23**, 5487-5493 (2021).
